# circRNAome profiling reveals circFgfr2 regulates myogenesis and muscle regeneration via a feedback loop

**DOI:** 10.1002/jcsm.12859

**Published:** 2021-11-22

**Authors:** Junyu Yan, Yalan Yang, Xinhao Fan, Guoming Liang, Zishuai Wang, Jiju Li, Liyuan Wang, Yun Chen, Adeyinka Abiola Adetula, Yijie Tang, Kui Li, Dazhi Wang, Zhonglin Tang

**Affiliations:** ^1^ Shenzhen Branch, Guangdong Laboratory for Lingnan Modern Agriculture, Agricultural Genomics Institute at Shenzhen Chinese Academy of Agricultural Sciences Shenzhen China; ^2^ Genome Analysis Laboratory of the Ministry of Agriculture and Rural Affairs, Agricultural Genomics Institute at Shenzhen Chinese Academy of Agricultural Sciences Shenzhen China; ^3^ Research Centre of Animal Nutritional Genomics, State Key Laboratory of Animal Nutrition, Institute of Animal Sciences Chinese Academy of Agricultural Sciences Shenzhen China; ^4^ Department of Cardiology, Boston Children's Hospital Harvard Medical School Boston MA USA; ^5^ GuangXi Engineering Centre for Resource Development of Bama Xiang Pig Bama China; ^6^ Kunpeng Institute of Modern Agriculture at Foshan Foshan China

**Keywords:** circRNA, circFgfr2, Skeletal muscle, Development, Regeneration, Feedback loop

## Abstract

**Background:**

Circular RNAs (circRNAs) represent a novel class of non‐coding RNAs formed by a covalently closed loop and play crucial roles in many biological processes. Several circRNAs associated with myogenesis have been reported. However, the dynamic expression, function, and mechanism of circRNAs during myogenesis and skeletal muscle development are largely unknown.

**Methods:**

Strand‐specific RNA‐sequencing (RNA‐seq) and microarray datasets were used to profile the dynamic circRNAome landscape during skeletal muscle development and myogenic differentiation. Bioinformatics analyses were used to characterize the circRNAome and identify candidate circRNAs associated with myogenesis. Bulk and single‐cell RNA‐seq were performed to identify the downstream genes and pathways of circFgfr2. The primary myoblast cells, C2C12 cells, and animal model were used to assess the function and mechanism of circFgfr2 in myogenesis and muscle regeneration *in vitro* or *in vivo* by RT‐qPCR, western blotting, dual‐luciferase activity assay, RNA immunoprecipitation, RNA fluorescence *in situ* hybridization, and chromatin immunoprecipitation.

**Results:**

We profiled the dynamic circRNAome in pig skeletal muscle across 27 developmental stages and detected 52 918 high‐confidence circRNAs. A total of 2916 of these circRNAs are conserved across human, mouse, and pig, including four circRNAs (circFgfr2, circQrich1, circMettl9, and circCamta1) that were differentially expressed (|log_2_ fold change| > 1 and adjusted *P* value < 0.05) in various myogenesis systems. We further focused on a conserved circRNA produced from the fibroblast growth factor receptor 2 (Fgfr2) gene, termed circFgfr2, which was found to inhibit myoblast proliferation and promote differentiation and skeletal muscle regeneration. Mechanistically, circFgfr2 acted as a sponge for miR‐133 to regulate the mitogen‐activated protein kinase kinase kinase 20 (Map3k20) gene and JNK/MAPK pathway. Importantly, transcription factor Kruppel like factor 4 (Klf4), the downstream target of the JNK/MAPK pathway, directly bound to the promoter of circFgfr2 and affected its expression via an miR‐133/Map3k20/JNK/Klf4 auto‐regulatory feedback loop. RNA binding protein G3BP stress granule assembly factor 1 (G3bp1) inhibited the biogenesis of circFgfr2.

**Conclusions:**

The present study provides a comprehensive circRNA resource for skeletal muscle study. The functional and mechanistic analysis of circFgfr2 uncovered a circRNA‐mediated auto‐regulatory feedback loop regulating myogenesis and muscle regeneration, which provides new insight to further understand the regulatory mechanism of circRNAs.

## Introduction

Skeletal muscle is the most abundant tissue in humans and other mammals of normal weight. The generation of skeletal muscle during embryonic development and postnatal regeneration relies on myogenesis,^1^, ^2^ which is a highly orchestrated process composed of the determination of multipotential mesodermal cells, myoblast proliferation and migration, fusion of myocytes into multinucleated myofibres, and maturation of the muscle fibre.^3^, ^4^ These processes are tightly and sophisticatedly controlled by the expression of myogenic genes such as myogenic regulatory factors (MRFs) and paired box (PAX) genes.^5^, ^6^, ^7^ Meanwhile, non‐coding RNAs (ncRNAs), such as microRNAs (miRNAs) and long non‐coding RNAs (lncRNAs), are emerging as regulators of skeletal muscle development and regeneration.^8^, ^9^, ^10^ For instance, our previous study suggested that miR‐148a promotes myogenic differentiation of both C2C12 myoblasts and primary muscle cells by targeting the Rho‐associated, coiled‐coil‐containing protein kinase 1 (ROCK1) gene.^9^

Circular RNAs (circRNAs) are a new type of endogenous ncRNA covalently closed by a non‐canonical splicing event called backsplicing.^11^, ^12^, ^13^ In recent years, owing to the development of high‐throughput RNA sequencing (RNA‐seq) and specific bioinformatics algorithms for circRNAs, thousands of circRNAs have been identified in eukaryotes including humans, mice, pigs, and chickens.^14^, ^15^, ^16^, ^17^, ^18^ Mounting evidence suggests that circRNAs play important roles in development and disease.^13^ To date, only a few circRNAs associated with myogenesis have been functionally and mechanistically characterized, and these typically act as molecular sponges for specific miRNAs, regulating the expression of muscle‐related genes via the competing endogenous RNA (ceRNA) mechanism.^19^, ^20^, ^21^, ^22^ For instance, circLMO7 regulates myoblast differentiation and survival by sponging miR‐378a‐3p.^19^ In addition to the sponge effect, other regulatory mechanisms of circRNAs in myogenesis have also been proposed. circZNF609, which is translated into protein in a splicing‐dependent and cap‐independent manner, controls myoblast proliferation.^23^ Despite these progresses, the dynamic expression of circRNAs during skeletal muscle development remains largely unclear and their roles in myogenesis and muscle regeneration require further systematic exploration.

The pig is an outstanding model organism for the study of human development and disease.^24^, ^25^, ^26^, ^27^ Recently, we generated the transcriptome landscape of skeletal muscle across 27 developmental stages using Ribo‐Zero strand‐specific RNA‐seq in pig.^28^ These datasets provide rich information for studying the function and regulation of non‐coding RNAs such as circRNA in skeletal muscle development. In the present study, with a view to systematically identifying circRNAs associated with myogenesis, we profiled the circRNAome landscape of skeletal muscle using these datasets and identified 52 918 high‐confidence circRNAs. Analysis of these circRNAs revealed that many are conserved across species and differentially expressed during skeletal muscle development and myoblast differentiation. Fibroblast growth factor receptor 2 (Fgfr2) is an important stimulatory modulator of satellite cells and is essential for the maintenance and repair of skeletal muscle.^29^ We further characterized one conserved circRNA produced from the Fgfr2 gene, termed circFgfr2, which was differentially expressed during skeletal muscle development and was up‐regulated during myogenic differentiation. Functional studies *in vitro* (mouse C2C12 cells and primary myoblasts) and *in vivo* revealed that circFgfr2 regulated myogenesis and muscle regeneration by acting as a sponge for miR‐133. We revealed a new Klf4‐mediated feedback loop between circFgfr2 and the JNK/MAPK pathway involving an exquisite mechanism for regulating myogenesis. Our findings provide a comprehensive circRNA resource for studying muscle development and demonstrate a circRNA‐mediated auto‐regulatory feedback loop regulating myogenesis.

## Materials and methods

### Identification of circRNAs in skeletal muscle

The Ribo‐Zero strand‐specific RNA‐seq data used for circRNA identification were obtained from our previous studies (GEO accession number GSE157044).^28^, ^30^ The transcriptome data were generated from the skeletal muscle of Landrace pigs at 27 developmental stages from embryonic day 33 (E33) to postnatal day 180 (D180). The corresponding whole‐genome bisulfite sequencing (WGBS) data (GEO accession number GSE157043) were used to evaluate the methylation level across the circRNAs. Further details on identification and characteristics analysis of circRNAs are provided in the Supporting Information, *Data* S1.

### Animal studies

C57BL/6 male mice were purchased from the Charles River Labs (Beijing, China) and maintained in our laboratory. All animal procedures were performed according to the protocols of the Chinese Academy of Agricultural Sciences and the Institutional Animal Care and Use Committee. No drug tests were carried out. For cardiotoxin (CTX) administration, adenoviruses expressing circFgfr2 (10^10^–10^11^ p.f.u./mL) were injected into the tibialis anterior (TA) muscles of 6‐week‐old C57BL/6 mice. After 28 days, the mice were injected with 100 μL CTX (10 μM) at the same place. Mice were sacrificed and TA muscles were collected at different time points after CTX injury. For further details, see *Data* S1.

### Cell isolation and culture

Mouse primary myoblasts, HEK293T and C2C12 cells, were used in this study. Mouse primary myoblasts were isolated from the TA muscles of newborn C57BL/6 mice. The primary myoblasts were cultured in Ham's F10 nutrient medium (Gibco) supplemented with 20% fetal bovine serum (Gibco) and 5 ng/mL basic fibroblast growth factor. HEK293T and C2C12 cells were cultured as previously described.^28^ For further details, see *Data* S1.

### Plasmid construction and RNA interference

To construct the circFgfr2‐overexpression vector, the full‐length sequence of mouse circFgfr2 was cloned into the pLCDH‐ciR vector (Geenseed Biotech, Guangzhou, China). To knock down circFgfr2, three siRNAs (si‐circFgfr2‐1, ‐2, and ‐3) targeting the back‐splicing junction (BSJ) of circFgfr2 and a negative control (siRNA‐NC) were synthesized (RiboBio Biotech, Guangzhou, China). The plasmid construction and siRNAs of other genes are described in *Data* S1.

### circRNA microarray analysis

The circRNA microarray was analysed using the Arraystar Mouse circRNA Array V2 (Arraystar, Rockville, MD, USA). After quantile normalization of the raw data, the differentially expressed circRNAs with statistical significance were identified using a Student's *t*‐test with a cut‐off value for false discovery rate (FDR) ≤ 0.05 and an absolute fold change (FC) ≥ 2. For further details, see *Data* S1.

### RNA‐sequencing analysis

The RNA‐seq libraries were prepared using the NEBNext Ultra RNA Library Prep Kit for Illumina (NEB, USA) according to the manufacturer's instructions and were sequenced on an Illumina NovaSeq platform to generate 150 bp paired‐end reads. The bioinformatics analysis of the RNA‐seq data was performed as described previously.^28^, ^30^ For further details, see *Data* S1.

### Single‐cell RNA‐sequencing analysis

Library construction and single‐cell RNA‐seq were performed according to the instructions of the Chromium Next GEM Single Cell 3 Reagent Kit v3.1. Sequencing reads were processed by Cell Ranger version 3.0.1 (10x Genomics, Pleasanton, CA, USA) using the mouse reference transcriptome mm10. Quality control, filtering, data clustering, data visualization, and differential expression analysis were performed using Seurat version 2.3.4 R package.^31^ For further details, see *Data* S1.

### Cell proliferation and cell cycle assay

Cell proliferation was assessed using the Cell‐Light EdU DNA cell proliferation kit (RiboBio) and Cell Counting Kit‐8 (CCK‐8) (Dojindo) according to the manufacturer's instructions. Flow cytometry analysis of the cell cycle was performed on a BD Accuri C6 flow cytometer (BD Biosciences, San Jose, CA, USA) as previously described,^18^ and data were processed using the FlowJo7.6 software (Treestar Incorporated, Ashland, OR, USA).

### RNA preparation and RT‐qPCR

Total RNA was extracted using TRIzol reagent (Invitrogen), and cDNAs were prepared using reverse transcriptase (Thermo Fisher Scientific). RT‐qPCR data were analysed using the ^ΔΔ^Ct method, and individual gene expression was normalized to GAPDH RNA expression. The primer sequences used in the present study are listed in *Table* S1. For further details, see *Data* S1.

### Western blotting and chromatin immunoprecipitation

Western blotting was performed as described previously.^30^, ^32^ Band intensities were quantified using the ImageJ program, normalized relative to the quantity of their respective internal bands, and expressed as percentages of the strongest value. The following dilutions were used for each antibody: myogenin(1:1000; ProteinTech), MyHC1 (1:1000; DSHB), Ki67 (1:1000; Abcam), MyoD (1:1000; ProteinTech), Pax7 (1:1000; ProteinTech), GAPDH (1:1000; ProteinTech), tubulin (1:1000; ProteinTech), MKK7 (1:1000; CST), p‐MKK7 (1:1000; CST), JNK (1:1000; CST), and p‐JNK (1:1000; CST). Chromatin immunoprecipitation (ChIP) was performed using a ChIP Assay Kit (EMD Millipore Corporation, Billerica, MA, USA) as described previously.^28^

### Other molecular experiments

The procedures used for histology, RNA‐FISH, immunohistochemistry, immunostaining RNA immunoprecipitation (RIP), and other assays are described in the *Data* S1.

### Data availability

All microarray expression, and RNA‐seq and scRNA‐seq data have been deposited in the China National GenBank (CNGB; https://db.cngb.org/) Nucleotide Sequence Archive (CNSA) under accession number CNP0001431.

## Results

### CircRNAome profiling of skeletal muscle across 27 developmental stages

**Figure 1:**
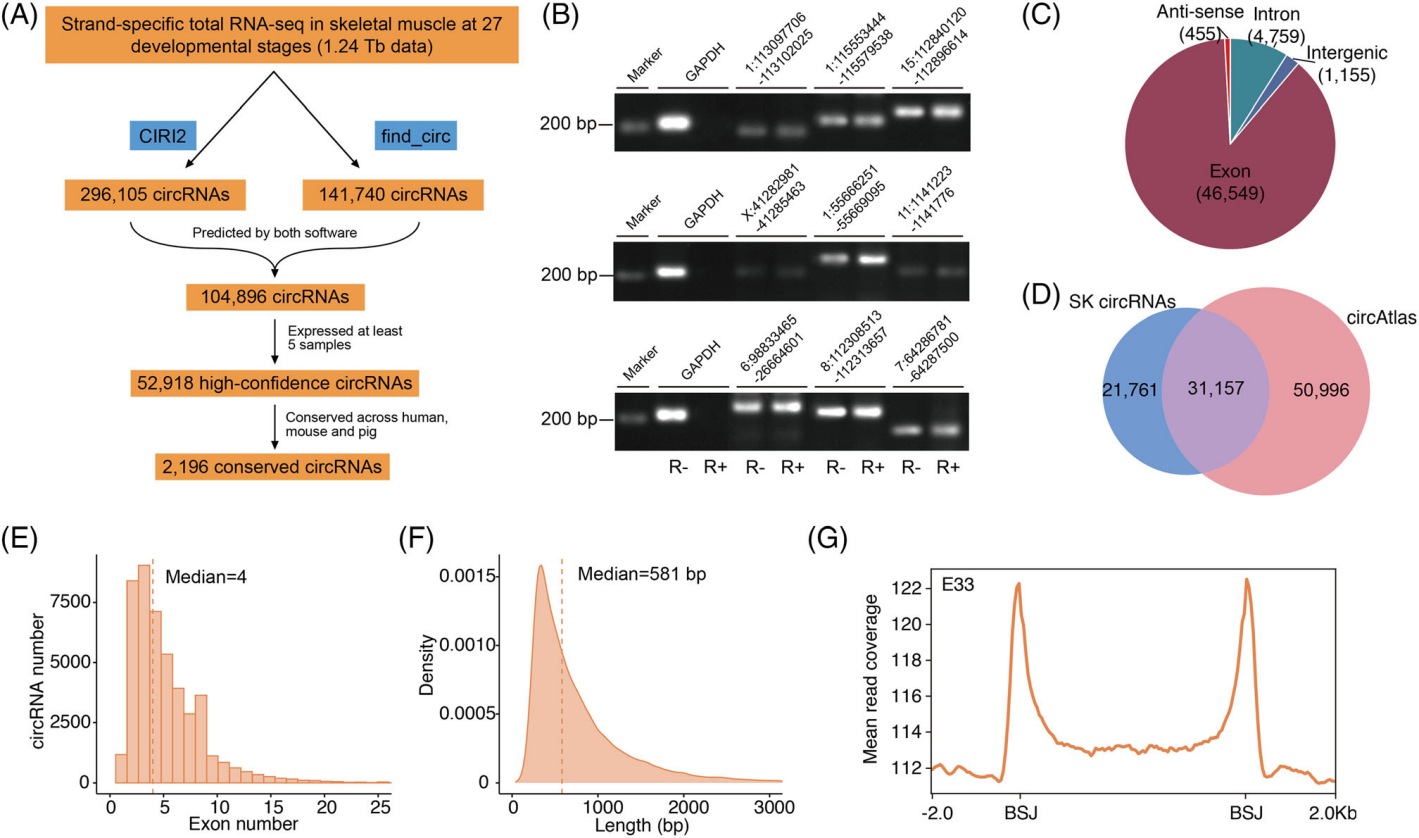
Identification and characteristics of circRNAs in pig skeletal muscle. (*A*) Pipeline for the identification of novel circRNAs. (*B*) Validation of the circularity of circRNAs. RT–PCR to detect circRNAs was performed on mock‐treated and RNase R‐treated RNA using divergent primers. Linear GAPDH mRNA was used as a negative control. (*C*) Distribution of circRNAs in different genomic regions. (*D*) Venn diagram showing the overlap between circRNAs that we identified and pig circRNAs deposited in the circAltas database. (*E*) The exon number distribution of identified circRNAs. (*F*) The length distribution of identified circRNAs. (*G*) Average read coverage of DNA methylation across gene bodies and the 2 kb regions flanking the BSJ sites of circRNAs at the E33 stage.

To characterize the landscape of circRNA profiles in skeletal muscle, we analysed an extensive RNA‐sequencing (RNA‐seq) dataset (GSE157044) that covers the 27 developmental stages of skeletal muscle and annotated a total of 104 896 circRNAs, which contained at least 2 unique back‐spliced reads. CircRNAs were considered robustly expressed when detected in a minimum of five distinct samples, resulting in a total of 52 918 high‐confidence circRNAs for downstream analysis (*Figure* 1A, *Table* S2). To validate the reliability of these circRNAs, 45 were chosen and confirmed by PCR using reverse transcription (RT–PCR) with divergent primers. BSJ was successfully detected in 43 of 45 (95.56%) circRNA candidates and confirmed by Sanger sequencing (*Table* S3). RT–PCR of the total RNase R‐treated RNA showed that these circRNAs were resistant to digestion, validating the circularity of circRNAs (*Figures* 1B and S1).

Most circRNAs were located in exons (46 549), followed by intronic (4759), intergenic (1155), and antisense (455) circRNAs (*Figure* 1C). In comparison with the known pig circRNAs reported in the circAltas database,^33^ we found that 41.12% (21 761/52 918) of the identified circRNAs were novel, which greatly expands the catalogue of pig circRNAs (*Figure* 1D, *Table* S3). Characteristics analysis showed that the median exon number and length of the exonic circRNAs were 4 (*Figure* 1E) and 581 bp (*Figure* 1F), respectively, similar to previous reports in pigs.^16^ Meanwhile, 72.76% (5312/7301) of the host genes corresponding to exonic circRNAs could produce at least 2 circRNAs (*Figure* S2A). Although the expression of most circRNAs was restricted to a subset of samples, we observed a subset of circRNAs (*n* = 3552) that was widely expressed (in at least 80% samples) in skeletal muscle (*Figure* S2B). We noticed that the gene body of circRNAs had a higher methylation level than that of neighbouring regions. Interestingly, we observed a marked hypermethylation around BSJ sites (*Figures* 1G and S3). We subsequently identified 12 677 and 4792 conserved circRNAs between pig and human and between pig and mouse, respectively. Among them, 2916 circRNAs were conserved across pig, human, and mouse (*Table* S2).

### Dynamic temporal regulation of circRNAs during skeletal muscle development

**Figure 2:**
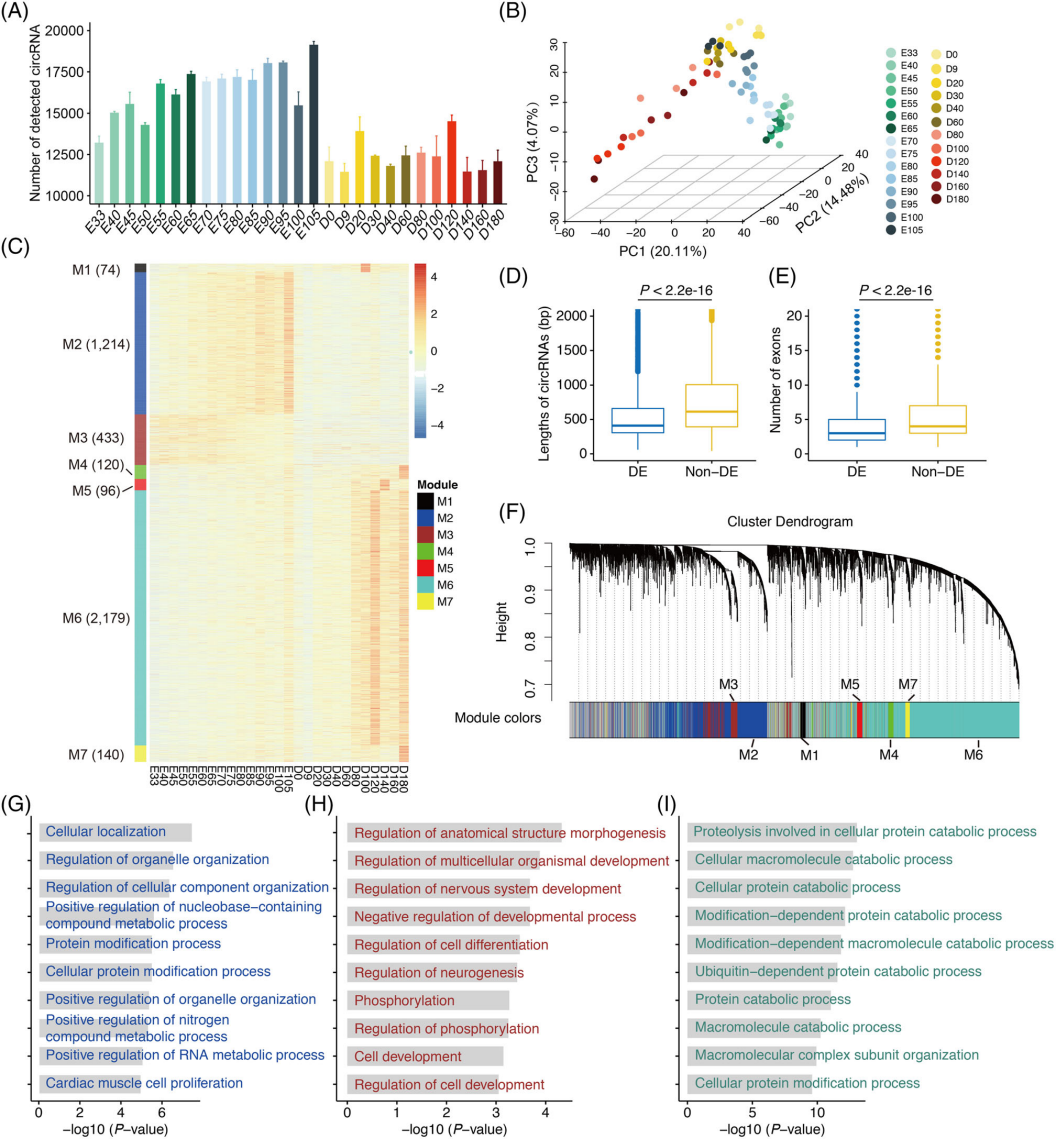
Dynamic expression of circRNAs during pig skeletal muscle development. (*A*) The number of detected circRNAs during each developmental stage. (*B*) PCA plot showing the global view of dynamic circRNA expression during skeletal muscle development, coloured according to developmental stage. The 3382 circRNAs that were expressed in at least 80% of samples were used for PCA analysis. (*C*) Heatmap showing the expression pattern of differentially expressed circRNAs (DECs) during skeletal muscle development. (*D*) Comparison of the transcript length between DECs and non‐DECs. (*E*) Comparison of the exon number between DECs and non‐DECs. (*F*) Dendrogram from WGCNA co‐expression network analysis of skeletal muscle samples. Modules of co‐expressed genes were assigned a colour and number (M1–M12). (*G*–*I*) GO enrichment analysis of the host genes of DECs in the M2 (*G*), M3 (*H*), and M6 (*I*) modules. The top 10 biological processes reported by DAVID 6.8 are shown.

We identified 9848–19 506 circRNAs expressed in each skeletal muscle, with many more circRNAs being expressed in prenatal than postnatal muscle (*Figure* 2A). Cluster analysis based on the expression of circRNAs clearly classified the skeletal muscle samples into prenatal and postnatal groups (*Figure* S4). Principal components analysis (PCA) revealed the overall pattern of circRNA expression was highly developmentally dependent, being ordered from embryonic to adult stages during skeletal muscle development (*Figure* 2B). These results suggest that circRNAs were dynamically expressed in a temporally dependent manner during skeletal muscle development.

To detect circRNAs that were temporally regulated during skeletal muscle development, a total of 7198 differentially expressed circRNAs (DECs) were identified between any two stages (log_2_ FC ≥ 1 and FDR ≤ 0.05; *Table* S4), accounting for 13.60% (7198/52 918) of all expressed circRNAs (*Figure* 2C). In comparison with exonic non‐DECs, exonic DECs had a shorter transcript length (*Figure* 2D) and a lower exon number (*Figure* 2E). Subsequently, we performed WGCNA co‐expression network analysis^34^ focusing on these DECs and revealed seven distinct co‐expression modules (M1–M7; *Table* S4). Three clusters (M2, M3, and M6) showed dynamic temporal expression during skeletal muscle development (*Figure* 2F). The circRNAs in clusters M2 and M3 were abundantly expressed during the prenatal stages. The expression of circRNAs in M2 gradually increased with skeletal muscle development and those in M3 gradually decreased. Gene ontology (GO) enrichment analysis showed that the host genes in M2 were enriched for GO categories related to cellular localization and proliferation (*Figure* 2G). The host genes in M3 were functionally involved in embryonic morphogenesis, nervous system development, and cell differentiation (*Figure* 2H). The circRNAs in M6 were mainly expressed during postnatal stages and up‐regulated during postnatal skeletal muscle development. These host genes of circRNAs in M6 were significantly associated with protein catabolic and modification processes (*Figure* 2I).

### circFgfr2 is highly conserved and a candidate regulator of skeletal muscle development

**Figure 3:**
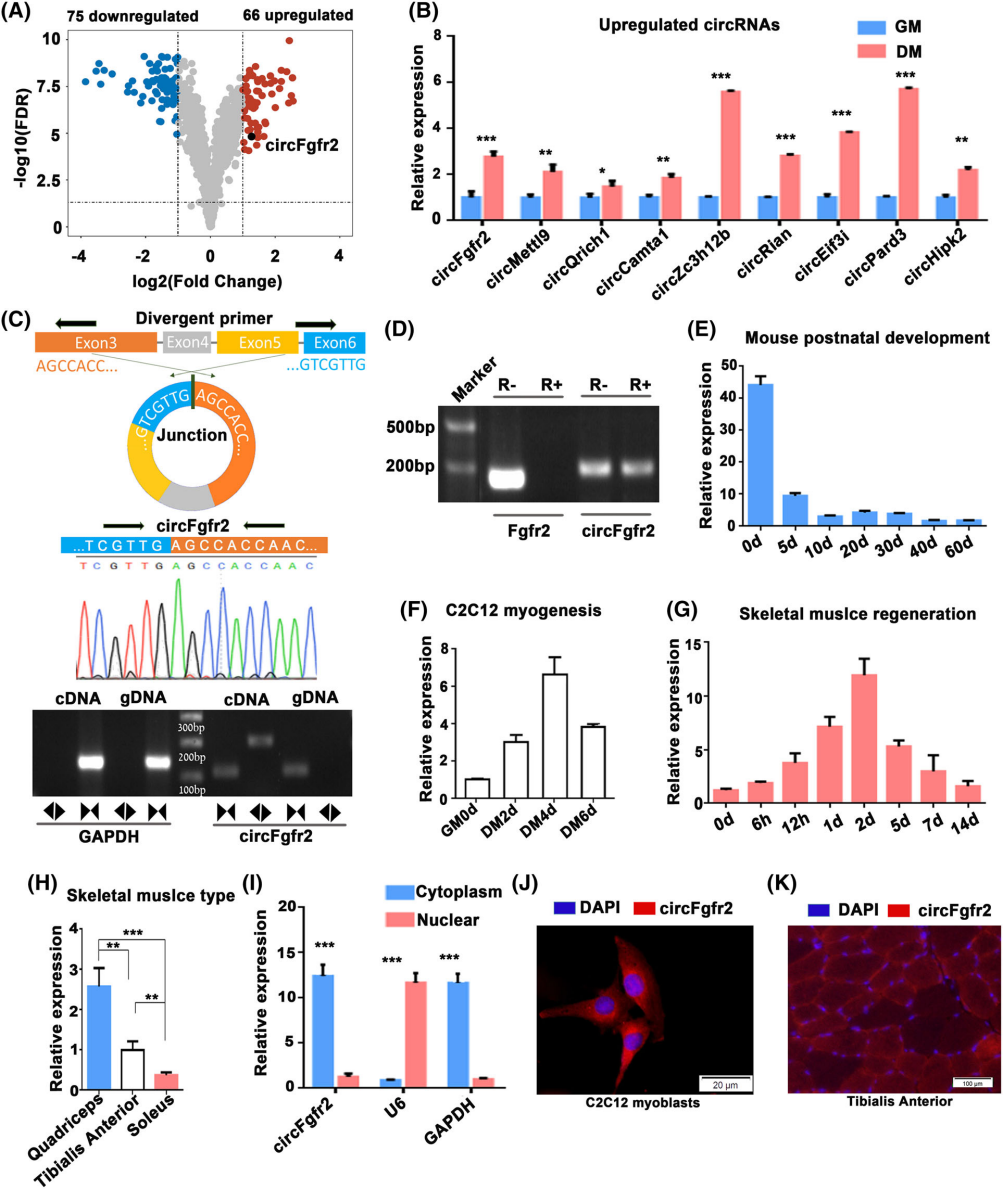
circFgfr2 is highly conserved and a candidate regulator of skeletal muscle development. (*A*) Volcano plot presents the differentially expressed circRNAs (|log_2_ fold change| > 1 and FDR < 0.05) in differentiated C2C12 myotubes (DM) as compared with myoblasts (GM). (*B*) Validation of up‐regulated circRNAs in DM by RT‐qPCR. (*C*) The BSJ of circFgfr2 was identified using a divergent primer and sequenced by Sanger sequencing. (*D*) RT–PCR analysis of circFgfr2 and linear Fgfr2 mRNAs on mock‐treated and RNase R‐treated RNA. (*E*–*G*) RT‐qPCR analysis of the expression of circFgfr2 during postnatal development in TA muscles from C57BL/6 mice (*E*), during C2C12 cell differentiation (*F*), and during CTX‐induced regeneration (*G*). (*H*) The expression level of circFgfr2 in different fibre types. The expression level was normalized to 18s‐ribosomal RNA. *N* = 3–5 in each group. (*I*) Determination of circFgfr2 localization by subcellular fractionation. The error bars depict the mean ± SD of samples from three to five individuals. **P < 0.01 and ***P < 0.001. (*J* and *K*) An RNA‐FISH assay was performed to determine circFgfr2 subcellular localization in C2C12 myoblasts (*J*) and TA muscles (*K*). Blue indicates nuclei stained with DAPI; red indicates the RNA probe that recognizes circFgfr2.The scale of (*J*) is 20 μm; (*K*) is 100 μm.

To further identify potential circRNAs involved in myogenesis, microarray analysis was carried out in mouse C2C12 myoblasts cultured in growth medium (GM) and in differentiation medium (DM). In comparison with myoblasts cultured in GM, 66 circRNAs were up‐regulated in myoblasts cultured in DM (log_2_ FC ≥ 1 and FDR ≤ 0.05; *Figure* 3A and *Table* S5). Six up‐regulated circRNAs were successfully validated by RT‐qPCR using divergent primers (*Figure* 3B), suggesting that these DECs were reliable for further analysis. Among the 66 circRNAs, six were identical to the pig circRNAs, including four circRNAs (circFgfr2, circQrich1, circMettl9, and circCamta1) that were also differentially expressed during pig skeletal muscle development, indicating that these circRNAs may regulate mammalian skeletal muscle development in a conserved manner.

Among these four conserved DECs, circFgfr2 aroused our interest. The host gene of circFgfr2, fibroblast growth factor receptor 2 (Fgfr2), is essential for the maintenance and repair of skeletal muscle.^29^ circFgfr2 is a stable exonic circRNA generated by exons 3–6 of the linear sequence in mice. The BSJ sequence of circFgfr2 was verified by Sanger sequencing (*Figure* 3C), and RT–PCR of circFgfr2 from RNase R‐treated RNA showed that circFgfr2 was resistant to digestion, indicating a closed‐loop structure (*Figure* 3D). Conservation analysis suggested that the BSJ sequences of circFgfr2 were highly conserved across humans, mice, pigs, and chickens (*Figure* S5).

We examined the temporal expression patterns in several myogenesis systems. The expression of circFgfr2 in mouse TA muscles was abundant during the neonatal stage and down‐regulated during postnatal development (*Figure* 3E). During C2C12 cell differentiation, the expression of circFgfr2 was found to be increased in a time‐dependent manner during the first 4 days following differentiation (*Figure* 3F). Meanwhile, using a well‐established CTX‐induced skeletal muscle damage and regeneration model (*Figure* S6), we found that the expression of circFgfr2 was highly induced during the first 2 days post‐injury and subsequently decreased (*Figure* 3G). However, the temporal expression patterns of circFgfr2 were independent of the patterns of Fgfr2 (*Figure* S7). Meanwhile, RT‐qPCR analysis revealed that circFgfr2 was enriched in fast‐twitch quadriceps muscle instead of slow‐twitch soleus muscle (*Figure* 3H), suggesting that the differential expression of circFgfr2 in different fibre types of skeletal muscles might depend on their metabolic status. Subcellular localization analysis based on FISH and chromatin fractionation experiments reveal that circFgfr2 was mainly distributed in the cytoplasm (*Figure* 3I–3K). Taken together, these data indicate that circFgfr2 is a potential circRNA regulating myogenesis and muscle regeneration.

### circFgfr2 represses myoblast proliferation and promotes myoblast differentiation and muscle regeneration

To explore the functions of circFgfr2 in myogenesis, we first investigated whether circFgfr2 affects the proliferation and differentiation of mouse myoblasts. Mouse primary myoblasts were transfected with a circFgfr2‐overexpression vector and siRNAs against circFgfr2. The overexpression vector (plCDH‐circFgfr2) and all three siRNAs (si‐circFgfr2‐1, ‐2, and ‐3) could efficiently change the expression of circFgfr2 (*Figure* S8), with no significant effect in the expression of linear *Fgfr2* mRNA (*Figure* S8C and S8F). si‐circFgfr2‐1 had the highest interference efficiency among the three siRNAs and was chosen for subsequent analysis (*Figure* S8E).

**Figure 4:**
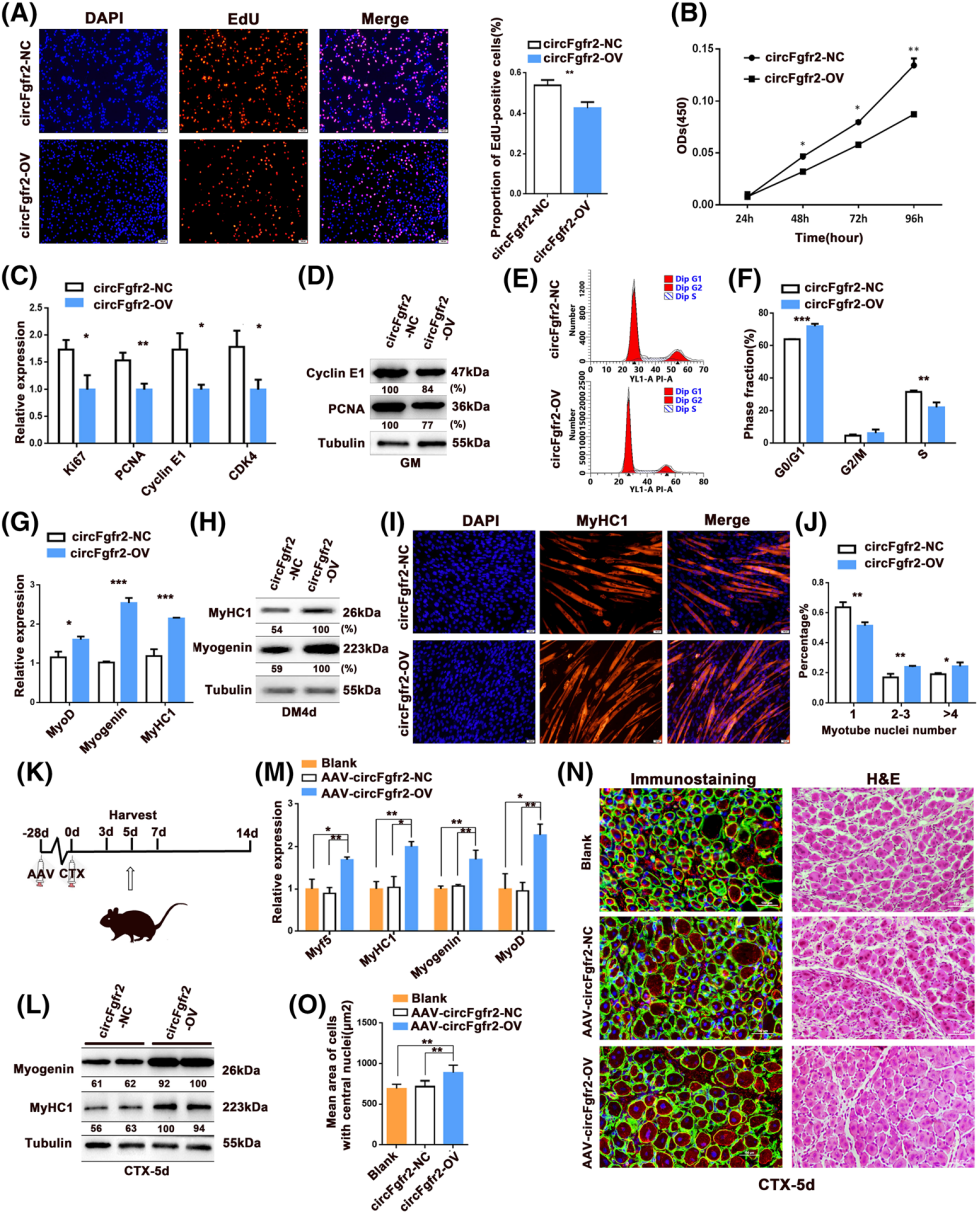
circFgfr2 represses myoblast proliferation and promotes myoblast differentiation and muscle regeneration. (*A*) EdU assay to assess cell proliferation after transfection with the circFgfr2‐overexpression vector (circFgfr2‐OV) and negative control (circFgfr2‐NC) in proliferating mouse primary myoblasts. Cell proliferation indices were assessed after treatment with EdU and counted using ImageJ. EdU staining (red) for positive cells; DAPI staining (blue) for cell nuclei. (*B*) Cell proliferation was assessed using the CCK‐8 assay after transfection with circFgfr2‐OV or circFgfr2‐NC vectors in proliferating mouse primary myoblasts. (*C* and *D*) The expression of proliferation and cell cycle markers was quantitated by RT‐qPCR (*C*) and western blotting (*D*) in proliferating mouse primary myoblasts. (*E* and *F*) The cell cycle was analysed using flow cytometry after transfection with circFgfr2‐OV and their negative controls in proliferating mouse primary myoblasts. The error bars depict the mean ± SD of samples from three individuals. (*G* and *H*) Following transfection of the circFgfr2‐OV and their negative controls (circFgfr2‐NC), the expression levels of myogenic differentiation markers MyoD, myogenin, and MyHC1 mRNA were detected by RT‐qPCR (*G*) and western blotting (*H*) in mouse primary myoblasts that differentiated after 4 days. (*I*) Immunofluorescence microscopy analysis of expression of MyHC1 after transfection with circFgfr2‐OV and circFgfr2‐NC in mouse primary myoblasts that differentiated after 4 days *in vitro*. The scale bars represent 100 μm. (*J*) The number of nuclei per myotube was counted after transfection with circFgfr2‐OV and circFgfr2‐NC in mouse primary myoblasts that differentiated after 4 days. (*K*) Injection scheme for AAV‐circFgfr2‐OV or AAV‐circFgfr2‐NC into CTX‐injured muscles. *N* = 5 per group. (*L*) RT‐qPCR analysis of the expression of Myf5, myogenin, MyHC1, and MyoD in AAV‐circFgfr2‐OV and AAV‐circFgfr2‐NC TA muscles on day 5 post‐injury. Data are presented as the mean ± SEM. (*M*) Western blotting of the expression of myogenin and MyHC1 in AAV‐circFgfr2‐OV and AAV‐circFgfr2‐NC TA muscles on day 5 post‐injury. (*N*) Immunostaining (left) and H&E staining (right) for desmin (red) and laminin (green) in AAV‐circFgfr2‐OV and AAV‐circFgfr2‐NC TA muscles on day 5 post‐injury (scale bar: 100 μm). (*O*) Average area of the cross‐sections of regenerating fibres on day 5 post‐CTX injury. *N* = 5 per group. The error bars depict the mean ± SD of samples from three to five replicates. *P < 0.05 and **P < 0.01.

In mouse primary myoblasts and C2C12 myoblasts, both the ethynyl‐2′‐deoxyuridine (EdU) incorporation assay and CCK‐8 assay showed that overexpression of circFgfr2 decreased cell proliferation activity (*Figures* 4A, 4B, and S9), and the opposite effect was observed following knockdown of circFgfr2 (*Figure* S9). Overexpression of circFgfr2 significantly decreased the expression of proliferation markers at both the mRNA and protein levels (*Figure* 4C and 4D), whereas knockdown of circFgf2 significantly up‐regulated the expression of these genes (*Figure* S9G and S9H). Cell cycle analysis revealed that overexpression of circFgfr2 increased the number of cells that progressed to the G0/G1 phase and reduced the number of cells that progressed to the S phase (*Figure* 4E and 4F), and the opposite effect was observed after circFgfr2 knockdown (*Figure* S9I and S9J). Meanwhile, overexpression of circFgfr2 significantly promoted the myogenic differentiation as demonstrated by the increased expression of MyHC1, MyoD, and myogenin at both the mRNA (*Figure* 4G) and protein levels (*Figure* 4H), and the immunofluorescence assay confirmed that circFgfr2 overexpression facilitated MyHC expression (*Figure* 4I) and myotube formation. Quantitative analysis showed that in comparison with the control, the circFgfr2 overexpression group exhibited more myotubes with multiple myonuclei (*Figure* 4J). Conversely, myoblast differentiation was significantly inhibited following circFgfr2 knockdown (*Figure* S9K–S9N).

Given that circFgfr2 was involved in myogenesis (*Figure* 3E–3G), we hypothesized that circFgfr2 also exerted a role in muscle regeneration *in vivo*. To validate this, TA muscles of C57BL/6 mice were injected with an adenovirus‐mediated circFgfr2‐overexpression vector (AAV‐circFgfr2‐OV) and controls (AAV‐circFgfr2‐NC). As expected, the expression of circFgfr2 was significantly up‐regulated after 28 days as compared with the controls (*Figures* 4K and S10A–S10C). Subsequently, CTX was intramuscularly injected at the same place (*Figure* 4K), and after 3 days, H&E and immunofluorescence staining of muscle cross‐sections showed a significantly lower amount of fibrosis/necrosis in AAV‐circFgfr2‐OV mice (*Figure* S10D). AAV‐circFgfr2‐OV suppressed the accumulation of mononucleated cells in the damaged area (*Figure* S10D). Meanwhile, we observed that the expression of MyoD and Pax7 was significantly higher in the AAV‐circFgfr2‐OV group as compared with that in the AAV‐circFgfr2‐NC group (*P* < 0.05; *Figure* S10E–S10G). Five days after CTX injection, new myofibres containing centralized nuclei were formed to repair damaged fibres, as expected (*Figures* 4L and S6). In comparison with the AAV‐circFgfr2‐NC group, AAV‐circFgfr2‐OV up‐regulated the expression of myogenin in TA muscle and stimulated the growth of new myofibres 5 days following CTX injection (*Figure* 4K and 4M). An increase in myofibre size was observed in muscle treated with AAV‐circFgfr2‐OV, whereas abundant mononucleated cells still existed in control muscle (*Figures* 4N and S10H). The cross‐sectional area of newly formed myofibres was significantly larger in the AAV‐circFgfr2‐OV group than that in the control muscle (*Figure* 4O). Taken together, these results suggest that circFgfr2 represses the proliferation of myoblasts and promotes myoblast differentiation and muscle regeneration.

### circFgfr2 regulates Map3k20 by sponging miR‐133

**Figure 5:**
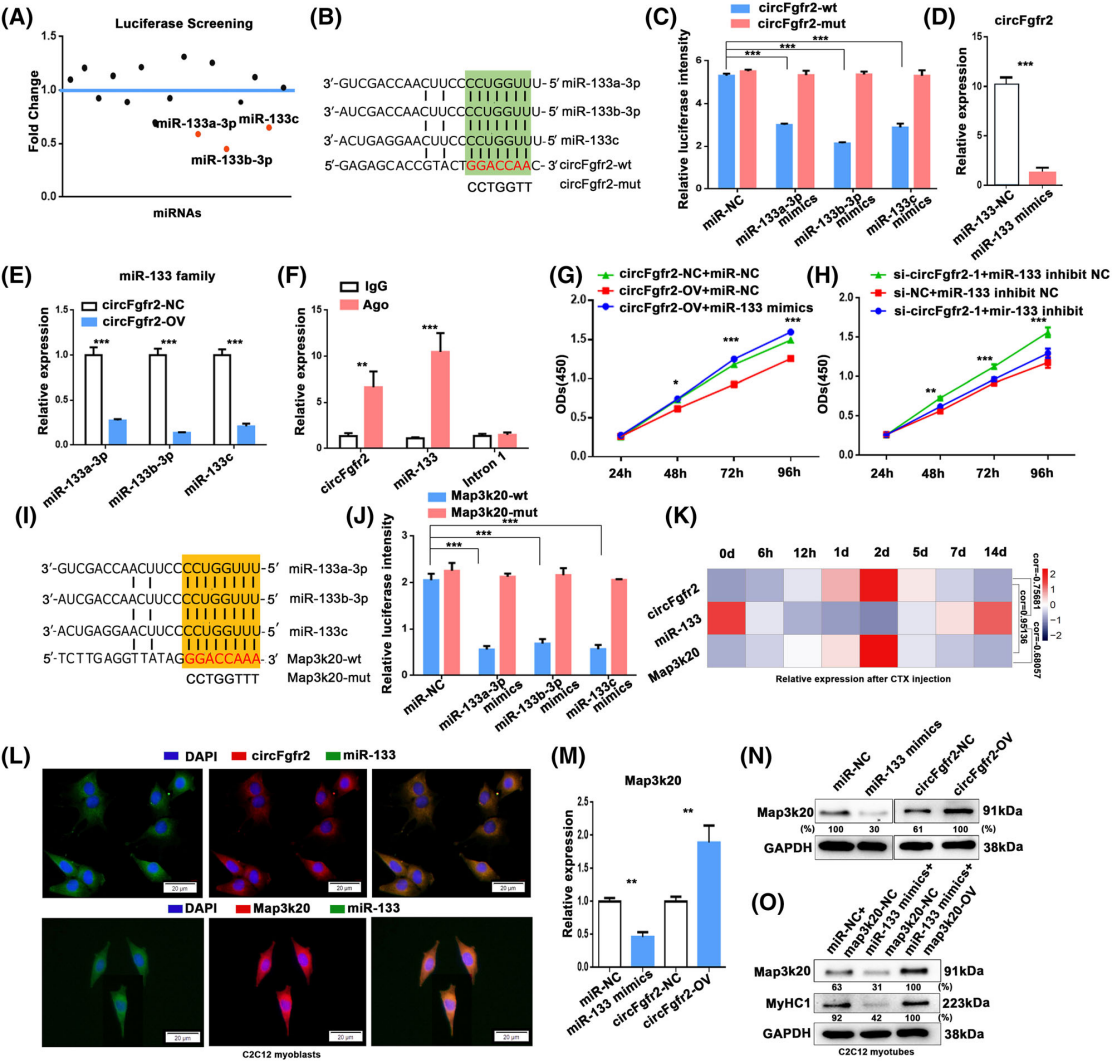
circFgfr2 acts as a ceRNA to promote Map3k20 expression by sponging miR‐133 family members. (*A*) Ratio of Firefly to Renilla luciferase activity following co‐transfection with each of 16 miRNA mimics and luciferase reporters containing all circFgfr2 sequences. (*B* and *C*) Ratio of Firefly to Renilla luciferase activity following co‐transfection with psicheck‐circFgfr2‐wt/psicheck‐circFgfr2‐mut and miR‐133 mimics/miR‐NC. (*D*) The effects of miR‐133 mimics on the expression level of circFgfr2 in C2C12 myoblasts indicated by RT‐qPCR. (*E*) RT‐qPCR showing the expression levels of miR‐133 family members following transfection with circFgfr2‐OV. (*F*) Fold enrichment of miR‐133 and circFgfr2 quantitated by RT‐qPCR after the RIP assay with the Ago2 antibody. IgG was used as a negative control for immunoprecipitation, and a sequence snippet from Fgfr2 intron 1 was used as a negative‐control region. (*G*) Effect of miR‐133 mimics or their negative control on circFgfr2‐OV inhibition of cell proliferation in C2C12 myoblasts by the CCK‐8 assay. (*H*) Effect of miR‐133 inhibitors and their negative control on si‐circFgfr2‐1 promotion of cell proliferation in C2C12 myoblasts by the CCK‐8 assay. (*I*) Schematic illustration of the predicted binding sites for miR‐133 family members in the 3′UTR of Map3k20. (*J*) HEK293T cells were co‐transfected with Map3k20 3′UTR or 3′UTR‐mut constructs and the miR‐133 family mimics. Data were normalized to the Renilla luciferase activity. (*K*) Heatmap showing the relative expression of circFgfr2, Map3k20, and miR‐133 in TA muscles during regeneration after CTX injury. The expression levels of these genes were determined by RT‐qPCR. The expression correlation between any two genes were calculated by Pearson correlation coefficient and was shown on the right. (*L*) An RNA‐FISH assay was performed to determine miR‐133, Map3k20, and circFgfr2 subcellular localization in proliferating C2C12 myoblasts. Scale bar, 20 μm. (*M* and *N*) RT‐qPCR (*M*) and western blotting (*N*) of Map3k20 expression in proliferating C2C12 myoblasts transfected with miR‐133 mimics or circFgfr2‐OV and their negative controls. (*O*) The expression levels of Map3k20 and MyHC1 proteins in C2C12 myoblasts co‐transfected with miR‐133 mimics and/or the Map3k20‐overexpression vector were higher than those in C2C12 myoblasts transfected with miR‐133 mimics alone as shown by western blotting. The error bars depict the mean ± SD of samples from three to five measurements. *P < 0.05, **P < 0.01, ***P < 0.001.

To determine the regulatory mechanism of circFgfr2 in myogenesis and muscle regeneration, we first evaluated miRNAs that had potential binding sites in the circFgfr2 transcript, and 16 miRNAs were predicted by both the TargetScan^35^ and miRDB^36^ programs. A luciferase reporter assay was performed in HEK293T cells expressing each of 16 miRNA mimics to validate the interactions. We found that the three miR‐133 family members (miR‐133a‐3p, 133b‐3p, and 133c) were the top three miRNAs that significantly reduced the luciferase intensity (*Figure* 5A and 5C), while they did not inhibit luciferase activity of the reporters with mutated miR‐133 binding sites (*Figure* 5B and 5C). Meanwhile, overexpression of miR‐133 significantly reduced the expression of circFgfr2 (*Figure* 5D), and overexpression of circFgfr2 significantly decreased the abundance of miR‐133 (*Figure* 5E). Next, we conducted Argonaute‐2 (Ago2, a core protein of the RISC)‐based RIP, followed by RT‐qPCR to examine whether Ago2 could pull down circFgfr2 and miR‐133 in C2C12 cells. As expected, there was a significant enrichment of circFgfr2 and miR‐133 in the Ago2 pull‐down samples (*Figure* 5F), suggesting that circFgfr2 closely interacted with miR‐133 via the Ago2 protein. In addition, overexpression of miR‐133 could effectively improve the inhibitory effect of circFgfr2 overexpression on myoblast proliferation (*Figure* 5G), and interference with miR‐133 mimics inhibited the myoblast proliferation promoting effect of circFgfr2 knockdown (*Figure* 5H). These findings strongly suggest that circFgfr2 is a direct target of miR‐133 family members.

We then explored the mechanism through which the miR‐133 family and circFgfr2 regulates myogenesis. Map3k20 was a putative target of miR‐133 family members predicted by both the TargetScan^35^ and miRDB^36^ programs (*Figure* 5I). The luciferase reporter assay showed that overexpression of miR‐133 significantly repressed the luciferase activity of the wild‐type Map3k20 3′UTR reporter, whereas this repression was abolished when the corresponding binding sites were mutated (*Figure* 5J). During skeletal muscle regeneration, Map3k20 had a negative correlation with miR‐133 and a positive correlation with circFgfr2 expression (*P* < 0.05; *Figure* 5K). Subcellular localization analysis showed that both miR‐133 and Map3k20 were mainly distributed in the cytoplasm similar to circFgfr2 (*Figure* 5L). Meanwhile, overexpression of miR‐133 family members resulted in a decrease in the expression of Map3k20, while overexpression of circFgfr2 significantly up‐regulated the expression of Map3k20 (*Figure* 5M and 5N). We observed that overexpression of Map3k20 effectively reversed the inhibitory effect of miR‐133 mimics on the expression of MyHC1 (*Figure* 5O). In addition, consistent with the functions of circFgfr2 in myogenesis, knockdown of Map3k20 inhibited myoblast proliferation and promoted differentiation (*Figure* S11), whereas opposite effect was observed following Map3k20 overexpression (*Figure* S11). Collectively, these results show that circFgfr2 acts as an miR‐133 family sponge to promote Map3k20 expression during myogenesis and muscle regeneration.

### Bulk and single‐cell RNA‐seq reveal that circFgfr2 regulates the activity of the JNK/MAPK signalling pathway

**Figure 6:**
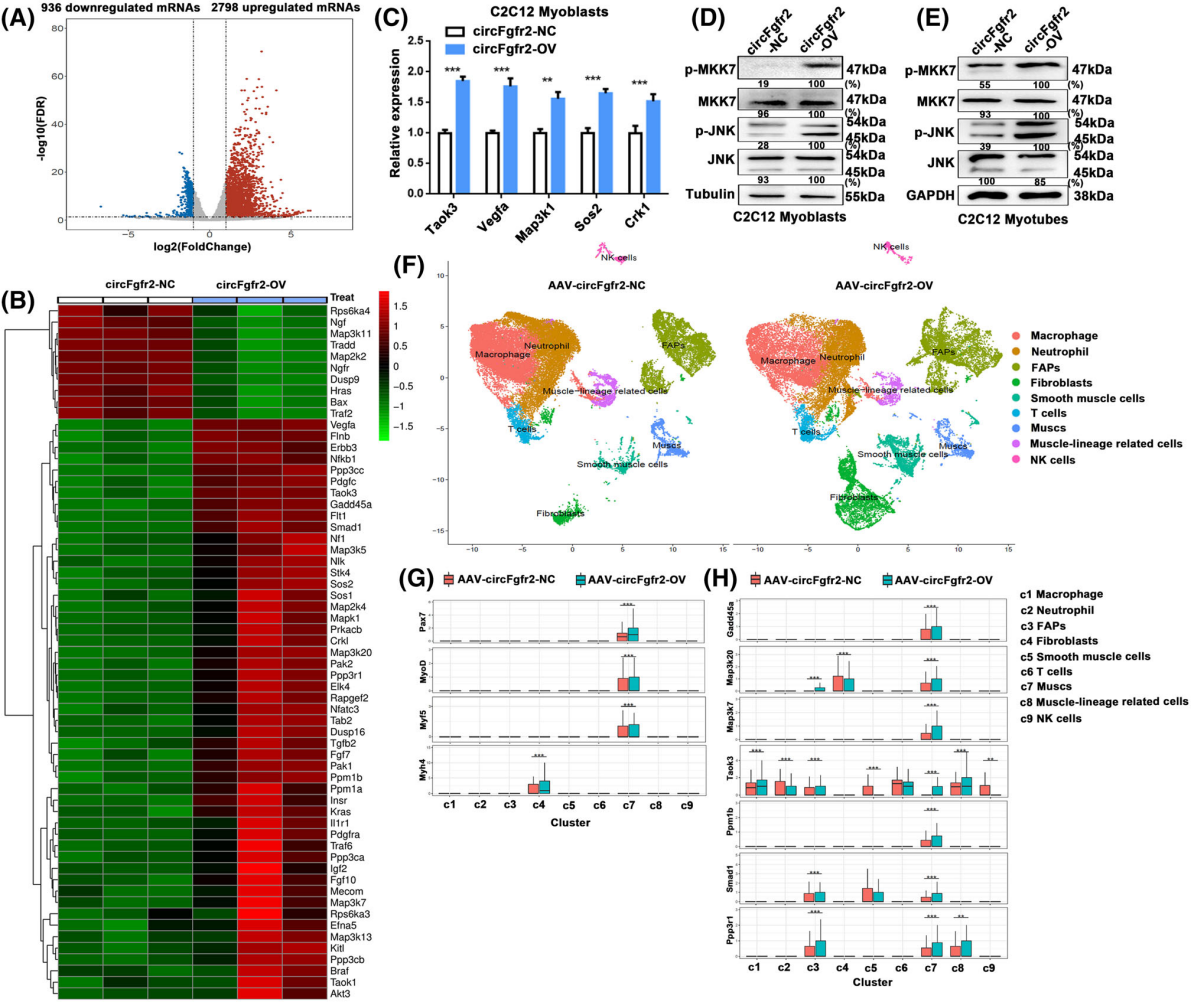
Bulk and single‐cell RNA‐seq reveal that circFgfr2 regulates the activity of the JNK/MAPK signalling pathway. (*A*) Volcano plot presents the differentially expressed mRNAs (|log_2_ fold change| > 1 and FDR < 0.05) in circFgfr2‐overexpressing C2C12 myoblasts as compared with their negative controls. (*B*) Heatmap analysis visualized the expression changes of genes in the MAPK signalling pathway following overexpression of circFgfr2. (*C*) Validation of differentially expressed mRNAs using RT‐qPCR. The error bars depict the mean ± SD of samples from three individuals. **P < 0.01 and ***P < 0.001. (*D* and *E*) Western blotting showing the expression of genes in the JNK/MAPK pathway following overexpression of circFgfr2 during the proliferation (*D*) and differentiation (*E*) stages. (*F*) Graph‐based clustering of FACS‐isolated single cells identifies distinct clusters corresponding to different skeletal muscle regeneration cell populations. (*G*) Expression pattern of MyoD, Myh4, Myf5, and Pax7 in skeletal muscle regeneration cell clusters. (*H*) The expression pattern of JNK pathway‐related genes in skeletal muscle regeneration cell clusters.

Map3k20 is one of the upstream factors regulating the JNK/MAPK signalling pathway.^37^ Given that circFgfr2 regulated Map3k20 expression by sponging miR‐133, we hypothesized that circFgfr2 may regulate skeletal muscle development via the JNK/MAPK signalling pathway. To address this, we first examined the global transcriptome changes by poly(A) RNA‐seq in proliferating C2C12 cells following the overexpression of circFgfr2. In comparison with the control group, 2798 genes were up‐regulated following circFgfr2 overexpression (*Figure* 6A, *Table* S6). Enrichment analysis suggested that the up‐regulated genes were significantly enriched for GO categories related to the cell cycle and KEGG pathways related to mitogen‐activated protein kinase (MAPK) signalling (*Figure* S12). Key genes of the MAPK signalling pathway were up‐regulated (*Figure* 6B). RT‐qPCR analysis validated that genes of the MAPK pathway were up‐regulated following circFgfr2 overexpression (*Figure* 6C). Meanwhile, western blotting analysis in C2C12 cells indicated that overexpression of circFgfr2 resulted in a marked increase in the expression of Map 3k20, p‐MKK7, and p‐JNK during both the proliferation (*Figure* 6D) and differentiation (*Figure* 6E) phases.

To further demonstrate the regulation of circFgfr2 in the JNK/MAPK pathway, we performed single‐cell RNA‐seq (scRNA‐seq) analysis (10× Genomics) in AAV‐circFgfr2‐OV‐treated muscle 5 days after CTX injection. After quality control, a total of 67 121 cells were retained (*Table* S7) and 9 cell types were identified by t‐SNE clustering and annotated (*Figure* 6F). Consistent with the enhancing function of circFgfr2 in muscle regeneration, the expression levels of MyoD, Myh4, Myf5, and Pax7 were elevated in muscle stem cells (MuSCs) from the AAV‐circFgfr2‐OV group as compared with those from the control group (*Figure* 6G). Meanwhile, overexpression of circFgfr2 activated the expression of Map3k20 and other key genes in the JNK pathway during muscle remodelling in muscle‐associated cell types (*Figure* 6H). These results indicate that circFgfr2 regulates myogenesis and muscle regeneration by activating the JNK/MAPK pathway.

### Klf4 targets circFgfr2 via a negative feedback loop

**Figure 7:**
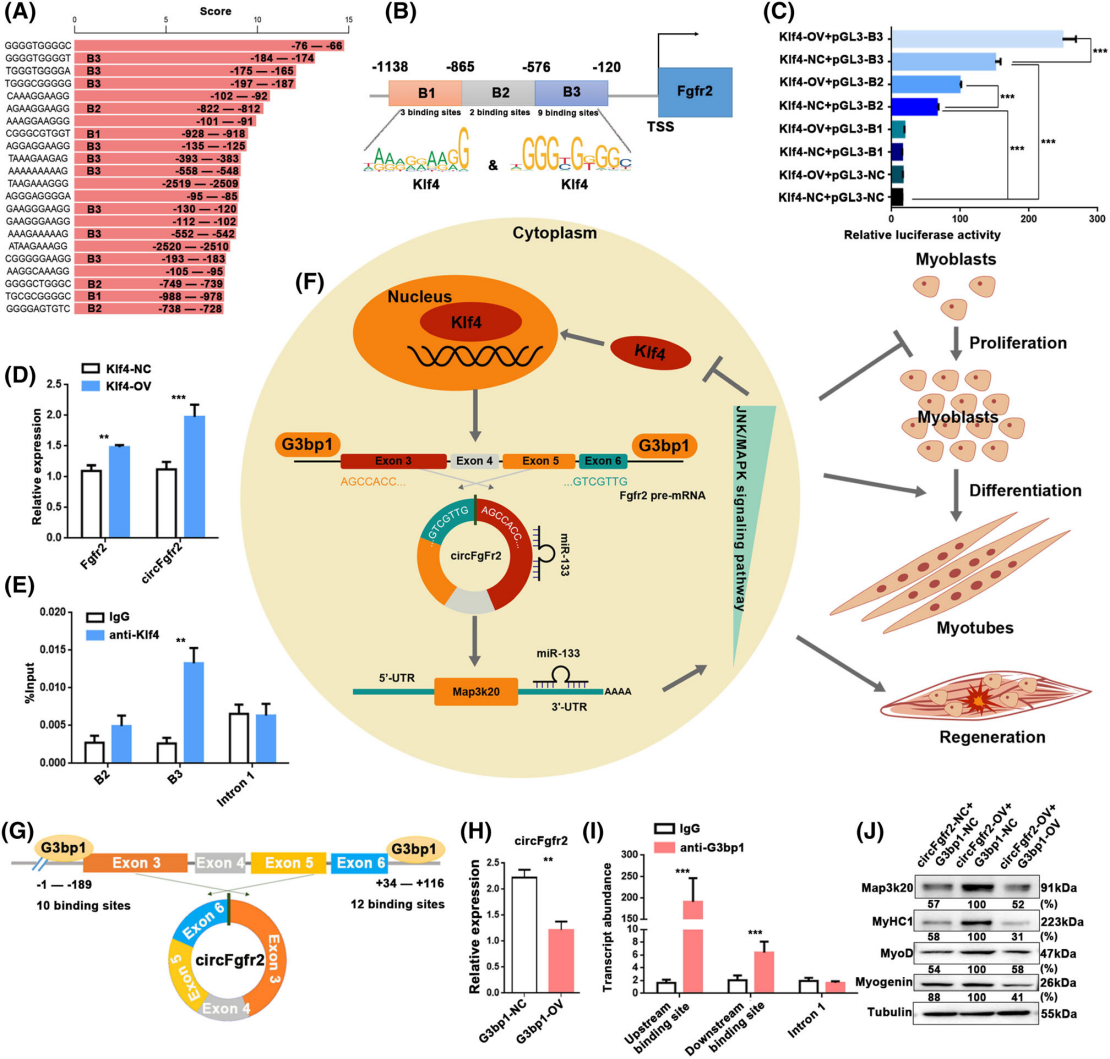
G3bp1 and Klf4 are upstream regulators of circFgfr2. (*A*) Enriched TF‐binding motif Klf4 in the Fgfr2 promoter. (*B*) Schematic illustration of the sequences of three putative binding regions of Klf4 in the Fgfr2 promoter are shown. (*C*) The relative luciferase activities were detected in HEK293T cells co‐transfected with luciferase reporter plasmids containing binding sites for the promoter sequence and overexpression plasmids of Klf4. HEK293T cells co‐transfected with empty pGL3‐Basic vector and empty pcDNA3.1 vector were used as negative controls. Firefly luciferase activity was normalized to Renilla luciferase activity. (*D*) The expression levels of Fgfr2 and circFgfr2 were detected in C2C12 myoblasts transfected with Klf4‐overexpression plasmids by RT‐qPCR. (*E*) ChIP‐qPCR was performed to determine which putative Klf4 binding site the Fgfr2 promoter was bound in C2C12 myoblasts. IgG was used as a negative control for immunoprecipitation, and a sequence snippet from Fgfr2 intron 1 was used as a negative‐control region. The error bars depict the mean ± SD of samples from three individuals. (*F*) Schematic diagram illustrating how the mechanism of circFgfr2 mediation by G3bp1 modulates skeletal muscle development via the miR‐133/Map3k20/JNK/MAPK/Klf4/circFgfr2 auto‐regulatory feedback loop. (*G*) The putative binding sites of G3bp1 in the upstream and downstream regions of circFgfr2 pre‐mRNA were predicted. (*H*) RT‐qPCR analysis of the expression of circFgfr2 following overexpression of G3bp1. (*I*) RIP confirmed that G3bp1 could directly bind to Fgfr2 pre‐mRNA in C2C12 myoblasts. IgG was used as a negative control for immunoprecipitation, and a sequence snippet from Fgfr2 intron 1 was used as a negative‐control region. (*J*) The expression levels of myogenic differentiation markers (MyoD, myogenin, and MyHC1) following co‐transfection of the G3bp1‐overexpression vector and/or circFgfr2‐OV were lower than those following transfection with circFgfr2‐OV alone, as shown by western blotting. In (*C*)–(*E*), (*H*), and (*I*), the error bars depict the mean ± SD of three replicates. *P < 0.05, **P < 0.01, ***P < 0.001.

Next, we predicted the transcription factors (TFs) that could potentially bind to the Fgfr2 promoter using JASPAR (http://jaspar.genereg.net). Among the predicted TFs, Klf4 caught our attention because it has been reported to promote muscle cell differentiation^38^ and skeletal muscle regeneration.^39^ There were multiple putative Klf4 binding motifs in the promoter of circFgfr2 (*Figure* 7A). We cloned three continuous regions containing these binding sites (B1–B3) and constructed a series of luciferase reporter vectors (*Figure* 7B). The results showed that Klf4 promoted the luciferase activity of the B2 and B3 promoter but did not affect the B1 promoter (*Figure* 7C). We constructed Klf4 overexpression vector and confirmed its effect at the protein level (*Figure* S13A). Overexpression of Klf4 significantly up‐regulated the expression of linear Fgfr2 mRNA and circFgfr2 (*Figures* 7D and S13A). Moreover, ChIP‐qPCR analysis demonstrated that Klf4 could bind to the B3 of the circFgfr2 promoter (*Figure* 7E). These data demonstrate that circFgfr2 is a direct target of Klf4.

Previous studies have shown that activation of JNK signalling and subsequent phosphorylation of Klf4 inhibits the Klf4 transcription and transactivation activity.^40^ Meanwhile, JNK/MAPK signalling pathway was reported to positively regulate myogenic differentiation.^41^ Accordingly, in conjunction with our findings, we proposed a circFgfr2‐mediated regulatory model of myogenesis in which circFgfr2 is regulated by Klf4 and acts as a decoy for miR‐133 to relieve its inhibitory effect on Map3k20 and activate the JNK/MAPK signalling pathway. Activation of JNK signalling inhibits Klf4 transcription and transactivation activity, eventually forming an auto‐regulatory feedback loop during myogenesis and muscle regeneration (*Figure* 7F).

### G3bp1 modulates the biogenesis of circFgfr2

To explore the mechanism that mediates the biogenesis of circFgfr2 during skeletal muscle development, we analysed the putative binding sites of RNA binding proteins (RBPs) in the canonical pre‐mRNA of circFgfr2. Six putative G3bp1 binding sites, two upstream and four downstream, were predicted (*Figure* 7G) via catRAPID.^42^ GTPase‐activating protein (SH3 domain) binding protein 1 (G3bp1) has been reported to function as an endoribonuclease that selectively targets genes by binding to their consensus sequence. RT‐qPCR showed that overexpression of G3bp1 significantly up‐regulated the expression of G3bp1 at the mRNA level as expected (*Figure* S13B) and down‐regulated the expression of circFgfr2, linear *Fgfr2* mRNA, and miR‐133 family in C2C12 myoblasts (*Figures* 7H, S13B, and S13C). RIP demonstrated that G3bp1 could bind to circFgfr2 pre‐mRNA via these putative upstream and downstream binding sites using an anti‐G3bp1 antibody (*Figure* 7I). Furthermore, western blotting showed that circFgfr2 overexpression promoted the expression of myoblast differentiation proteins and these effects could be reversed by co‐transfection of C2C12 cells with an G3bp1‐overexpression vector (*Figure* 7J). Therefore, G3bp1 binds directly to circFgfr2 and inhibits its biogenesis to modulate muscle development.

## Discussion

Accumulating evidence suggests that circRNAs are not simply junk‐products of pre‐mRNA splicing and points to the potential of circRNAs as novel regulators in a wide range of developmental processes and diseases.^11^, ^12^, ^13^ Here, we comprehensively profile the dynamic circRNAome atlas of skeletal muscle across 27 developmental stages and identified ~53 000 high‐confidence circRNAs in pigs. Approximately 40% of these circRNAs have not been previously identified, which substantially expands the catalogue of circRNAs in mammals.^33^

Previous studies have suggested that the function of circRNAs is conserved across species.^43^ In the present study, we observed that a large proportion of circRNAs are highly conserved across humans, pigs, and mice, suggesting that their functions in myogenesis may also be conserved. Remarkably, we observed that four circRNAs are also up‐regulated in differentiated C2C12 myotubes as compared with proliferating myoblasts, which are also differentially expressed during pig skeletal muscle development. Our analytical approach provides an efficient framework to identify conserved circRNAs regulating skeletal muscle development.

To further understand the function and regulatory mechanism of circRNAs during skeletal muscle development, we focused on circFgfr2, which is produced from the Fgfr2 gene. Fgfr2 is an important stimulatory modulator of satellite cells in skeletal muscle and plays a critical role in muscle growth and repair.^29^ A previous study in chickens has suggested that circFgfr2 promotes myoblast proliferation and differentiation.^18^ However, our results reveal that circFgfr2 inhibits myoblast proliferation and promotes differentiation of mouse myoblasts, suggesting that circFgfr2 may have different effects on myoblast proliferation in mammals and chickens. Our results indicate that circFgfr2 is a critical myogenesis regulator during skeletal muscle development and regeneration.

The most well‐known functions of circRNAs are as molecular sponges of specific microRNAs and regulators of gene expression via the ceRNA mechanism.^13^ Our results suggest that circFgfr2 acts as a decoy for miR‐133 through the ceRNA mechanism to affect the expression of Map3k20. miR‐133 is specifically expressed in muscle and has been reported to inhibit myoblast differentiation and promote proliferation.^44^ Map3k20 is a member of the MAPKKK family of signal transduction molecules, which forms homodimers to regulate the JNK/MAPK pathway.^37^ JNK/MAPK signalling pathway plays a positive role in regulating myogenic differentiation.^41^ We found that circFgfr2 can activate the JNK/MAPK signalling pathway. In addition, we confirmed that circFgfr2 is regulated by G3bp1 and Klf4. Klf4 belongs to the KLF family of evolutionarily conserved zinc finger TFs that regulate muscle cell differentiation and skeletal muscle regeneration.^38^, ^39^ Interestingly, previous studies have shown activation of JNK signalling and subsequent phosphorylation of Klf4 inhibits the Klf4 transcription.^40^ Therefore, we propose that circFgfr2 is regulated by Klf4 and acts as a ceRNA sponge of miR‐133 family members to activate the expression of Map3k20 and the downstream JNK/MAPK signalling pathway. Conversely, the activation of JNK signalling inhibits Klf4 transcription and transactivation activity, establishing an auto‐regulatory feedback loop (*Figure* 7F).

In summary, we profile the first dynamic circRNAome across 27 developmental stages in skeletal muscle and during myoblast differentiation. The present work not only provides a rich circRNA resource for the study of muscle but also outlines a framework for the high‐throughput identification of candidate circRNAs associated with skeletal muscle development. We deeply analyse the function and mechanism of conserved circFgfr2 and suggest a circRNA‐mediated auto‐regulatory feedback loop regulating myogenesis and muscle regeneration, which provides new insight to further understand the regulatory mechanism of circRNAs.

## Funding

This work was supported by the National Natural Science Foundation of China (31830090), the Basic and Applied Basic Research Foundation of Guangdong Province (2019B1515120059), the National Key Project (2016ZX08009‐003‐006), and the Agricultural Science and Technology Innovation Program (CAAS‐ZDRW202006).

## Conflict of interest

None declared.

## Supporting information


**Figure S1.** **Verification of the reliability of pig circRNAs.** Pig circRNAs were validated by PCR with reverse transcription (RT–PCR) using divergent primers following RNase R treatment.
**Figure S2. The production and expression of identified circRNAs. (A)** Number of circRNAs produced from one gene. **(B)** The distribution of the sample number per circRNA expressed.
Figure S3. Average read coverage of DNA methylation across gene bodies and the 2‐kb regions flanking the BSJ sites of circRNAs at each developmental stage, except E33.
**Figure S4. Hierarchical cluster analysis of the skeletal muscle samples.** Clustering was performed based on the log2‐transformed CPM values of 3,382 circRNAs that were expressed in at least 80% of samples using the average linkage method by the hclust function in R.
**Figure S5. Sequence alignment of the BSJ sequences of circFgfr2 in humans, mice, pigs, and chickens.** The BSJ sequences of circFgfr2 in humans (hsa‐Fgfr2_0001) were downloaded from the circAltas database. The BSJ sequence of chicken circFgfr2 was obtained from a previous study [32]. The BSJ sequences of mouse and pig circFgfr2 were amplified in the present study. Sanger sequencing was performed to validate the BSJ sequences of circFgfr2 in the four species.
**Figure S6. Construction of the muscle regeneration model following CTX injection in the tibialis anterior (TA)**. **(A)** H&E staining of the cross‐sections of CTX‐induced TA muscle. **(B‐E)** RT‐qPCR showing the expression levels of *myogenin*, *MyoD*, *MyHC1,* and *Pax7* during muscle regeneration. The expression level was normalized to 18s‐ribosomal RNA. *N* = 3–5 in each group.
**Figure S7. Expression patterns of Fgfr2 in mice**. (**A‐B**) The expression of Fgfr2 was quantitated by RT‐qPCR (**A**) and western blotting (**B**) during CTX‐induced TA muscle regeneration. (**C‐D**) RT‐qPCR (**C**) and western blotting (**D**) showing the expression levels of Fgfr2 during C2C12 myogenesis. (**E**) The expression of Fgfr2 was quantitated by RT‐qPCR during postnatal development in the hind leg muscles of C57BL/6 mice. The expression level was normalized to 18s‐ribosomal RNA. *N* = 3–5 in each group.
**Figure S8. The knockdown and overexpression of circFgfr2 in C2C12 cells and mouse primary myoblasts. (A)** Schematic diagram showing the construction of the circFgfr2‐overexpression (pLCDH‐circFgfr2) vector. **(B)** RT‐qPCR showing the overexpression efficiency of the circFgfr2‐overexpression vector in C2C12 cells. **(C)** RT‐qPCR showing the effects of circFgfr2 overexpression on the expression of circFgfr2 and host gene Fgfr2 in mouse primary myoblasts. The error bars depict the mean ± S.D. of samples from three individuals. ***P < 0.001. **(D)** Schematic diagram showing the design of three siRNA oligos against circFgfr2 (si‐circFgfr2‐1, ‐2, and ‐3). **(E)** RT‐qPCR showing the interference efficiency of the three siRNAs in C2C12 myoblasts. **(F)** RT‐qPCR showing the effect of si‐circFgfr2‐1 on the expression of circFgfr2 and Fgfr2 in mouse primary myoblasts. The error bars depict the mean ± S.D. of samples from three individuals. ***P < 0.001.
**Figure S9. Knockdown of circFgfr2 promotes proliferation but prevents differentiation. (A‐C)** Cell proliferation indices were assessed following treatment with 5‐ethynyl‐2′‐deoxyuridine (EdU) and counted using Image J, after transfection with si‐circFgfr2‐1 (**A**) and circFgfr2‐OV (**B**) in proliferating C2C12 myoblasts, and (**C**) si‐circFgfr2‐1 in proliferating mouse primary myoblasts. EdU staining (red) for positive cells; DAPI staining (blue) for cell nuclei. The error bars depict the mean ± S.D. of samples from three individuals. *P < 0.05 **(D‐F)** Cell counting kit‐8 (CCK‐8) assay showing the cell proliferation activity following transfection with si‐circFgfr2‐1 (**D**) or circFgfr2‐OV (**E**) in proliferating C2C12 myoblasts, and (**F**) si‐circFgfr2‐1 in proliferating mouse primary myoblasts. Data are presented as the mean ± S.D. of samples from four individuals. *P < 0.05, **P < 0.01, ***P < 0.001 **(G‐H)** Transfection of proliferating mouse primary myoblasts with si‐circFgfr2–1 and their negative controls. The proliferation and cell cycle markers were quantitated by RT‐qPCR (**G**) and western blotting (**H**). The error bars depict the mean ± S.D. of samples from three individuals. *P < 0.05 and **P < 0.01. **(I‐J)** The cell cycle was analyzed using flow cytometry following transfection with si‐circFgfr2‐1 and their negative controls in proliferating mouse primary myoblasts. The error bars depict the mean ± S.D. of samples from three individuals. *P < 0.05 and **P < 0.01. **(K‐L)** Transfection of mouse primary myoblasts with si‐circFgfr2‐1 and their negative controls. The mRNA and protein expression levels of myogenic differentiation marker genes (MyoD, myogenin, and MyHC1) were detected by RT‐qPCR (**K**) and western blotting (**L**) in mouse primary myoblasts that differentiated after 4 days in vitro, respectively. The error bars depict the mean ± S.D. of samples from three individuals. *P < 0.05 and **P < 0.01. (**M‐N)** Immunofluorescence analysis of MyHC1 myotubes (M) and the number of nuclei per myotube was counted (N) following knockdown of circFgfr2 in mouse primary myoblasts that differentiated after 4 days in vitro; the scale bars represent 100 μm.
**Figure S10. circFgfr2 promotes skeletal muscle regeneration. (A‐C)** RT‐qPCR (**A**), H&E staining (B) and fluorescence images (**C**) showing that injection of AAV‐circFgfr2‐OV increased circFgfr2 expression in mouse TA muscles 28 days after adenovirus injection. The expression level was normalized to 18s‐ribosomal RNA. ** *P* < 0.01 post‐injury vs. baseline level; *n* = 5 in each group. Data are presented as the mean ± SEM. **(D)** H&E staining, Sirius Red staining and immunostaining for desmin (red) and laminin (green) of AAV‐circFgfr2‐OV and AAV‐ circFgfr2‐NC TA muscles on day 3 post‐CTX injury (Scale bar: 50 μm in H&E staining, Sirius Red staining; 100 μm in immunostaining). (**E‐F**) RT‐qPCR revealing that expression of Map3k20, Pax7, and MyoD was dramatically upregulated and that of miR‐133 was downregulated in AAV‐circFgfr2‐OV mice on day 3 post‐CTX injury. The expression level was normalized to 18s‐ribosomal RNA. ** *P* < 0.01 post‐injury vs. baseline level; *n* = 3–5 in each group. Data are presented as the mean ± SEM. **(G)** Western blotting showing the expression of Map3k20, Pax7 and MyoD in AAV‐circFgfr2‐OV mice on day 3 post‐CTX injury. (**H**) Sirius Red staining of AAV‐circFgfr2‐OV and AAV‐ circFgfr2‐NC TA muscles on day 5 post‐CTX injury (Scale bar: 100 μm).
**Figure S11. Map3k20 promotes myoblast differentiation but inhibits proliferation in C2C12 cells. (A‐B)** RT‐qPCR showing the expression of Map3k20 during postnatal skeletal muscle development in mice (**A**) and during C2C12 myoblast myogenesis (**B**). **(C)** RT‐qPCR showing the expression of proliferation markers *(PCNA and Ki67)* and myogenic differentiation marker genes *(MyoD, myogenin, and MyHC1*) following transfection with the Map3k20‐overexpression vector in proliferating C2C12 cells. The error bars depict the mean ± S.D. of samples from three individuals. * *P* < 0.05, ** *P* < 0.01, *** *P* < 0.001. **(D)** RT‐qPCR showing the interference efficiency of three Map 3k20 siRNAs (si‐Map3k20‐1, ‐2, and ‐3) and their effect on the expression of Ki67 and PCNA in proliferating C2C12 myoblasts. The error bars depict the mean ± S.D. of samples from three individuals. * *P* < 0.05, ** *P* < 0.01, *** *P* < 0.001. **(E)** RT‐qPCR showing the expression levels of myogenic differentiation marker genes (*MyoD*, *myogenin*, and *MyHC1*) following transfection with si‐Map3k20‐1 in C2C12 cells that differentiated after 4 days *in vitro*. The error bars depict the mean ± S.D. of samples from three individuals. * *P* < 0.05, ** *P* < 0.01. **(F)** Western blotting showing the expression levels of PCNA and Cyclin E1 in proliferating C2C12 cells following transfection of Map3k20, si‐Map3k20‐1, and their negative controls. **(G)** EdU assay to assess cell proliferation after transfection with Map3k20‐OV or Map3k20‐NC in proliferating C2C12 myoblasts. Cell proliferation indices were assessed after treatment with EdU and counted using Image J. EdU staining (red) for positive cells; Dapi staining (blue) for cell nuclei. The scale bars represent 100 μm. **(H)** Western blotting showing the expression levels of myogenin and MyHC1 after transfection with Map3k20, si‐Map3k20‐1, and their negative controls in C2C12 cells that differentiated after 4 days *in vitro*. **(I)** Immunofluorescence microscopy analysis of expression of MyHC1 and the number of nuclei per myotube was counted in Map3k20‐overexpressing C2C12 cells that differentiated after 4 days *in vitro*. The scale bars represent 100 μm.
**Figure S12. GO and KEGG pathway analyses of differentially expressed genes in circFgfr2‐overexpressing circFgfr2 myoblasts as compared with the negative control. (A‐B)** GO (**A**) and KEGG (**B**) pathway analyses of upregulated genes. **(C‐D)** GO (**C**) and KEGG (**D**) pathway analyses of downregulated genes.
**Figure S13. Klf4 and G3bp1 modulates the transcription of circFgfr2.** (**A**)Western blotting showing the expression levels of Klf4 and Fgfr2 in C2C12 cells following transfection with Klf4‐OV or Klf4‐NC. (**B**) The expression level of G3bp1 and Fgfr2 in G3BP1‐overexpressing C2C12 cells. The error bars depict the mean ± S.D. of samples from three individuals. *** *P* < 0.001. **(C)** The expression level of miR‐133 family in G3BP1‐overexpressing C2C12 cells. The error bars depict the mean ± S.D. of samples from three individuals. ** *P* < 0.01, *** *P* < 0.001.

**Data S1.** Detail methods used in the present study.

**Table S1.** Information regarding the primers used in the present study.

**Table S2.** Identification of circRNAs in skeletal muscle.

**Table S3.** The circular junction of circRNAs was identified using divergent primers in Sanger sequencing.

**Table S4.** Differentially expressed circRNAs during skeletal muscle development in pigs.

**Table S5.** Differentially expressed circRNAs between differentiated C2C12 myotubes and proliferating myoblasts.

**Table S6.** Differentially expressed mRNAs in circFgfr2‐overexpressing C2C12 myoblasts as compared with their negative controls.

**Table S7.** Summary of the scRNA‐seq data.
