# Supplementary material for: circRNAome profiling reveals circFgfr2 regulates myogenesis and muscle regeneration via a feedback loop
Source: J Cachexia Sarcopenia Muscle. 2021 Nov 22;13(1):696–712. doi: 10.1002/jcsm.12859 (PMC8818660; doi:10.1002/jcsm.12859)
Supplement: Supplementary file 1 — Figure S1. Verification of the reliability of pig circRNAs. Pig circRNAs were validated by PCR with reverse transcription (RT–PCR) using divergent primers following RNase R treatment. Figure S2. The production and expression of identified circRNAs. (A) Number of circRNAs produced from one gene. (B) The distribution of the sample number per circRNA expressed. Figure S3. Average read coverage of DNA methylation across gene bodies and the 2‐kb regions flanking the BSJ sites of circRNAs at each developmental stage, except E33. Figure S4. Hierarchical cluster analysis of the skeletal muscle samples. Clustering was performed based on the log2‐transformed CPM values of 3,382 circRNAs that were expressed in at least 80% of samples using the average linkage method by the hclust function in R. Figure S5. Sequence alignment of the BSJ sequences of circFgfr2 in humans, mice, pigs, and chickens. The BSJ sequences of circFgfr2 in humans (hsa‐Fgfr2_0001) were downloaded from the circAltas database. The BSJ sequence of chicken circFgfr2 was obtained from a previous study [32]. The BSJ sequences of mouse and pig circFgfr2 were amplified in the present study. Sanger sequencing was performed to validate the BSJ sequences of circFgfr2 in the four species. Figure S6. Construction of the muscle regeneration model following CTX injection in the tibialis anterior (TA). (A) H&E staining of the cross‐sections of CTX‐induced TA muscle. (B‐E) RT‐qPCR showing the expression levels of myogenin, MyoD, MyHC1, and Pax7 during muscle regeneration. The expression level was normalized to 18s‐ribosomal RNA. N = 3–5 in each group. Figure S7. Expression patterns of Fgfr2 in mice. (A‐B) The expression of Fgfr2 was quantitated by RT‐qPCR (A) and western blotting (B) during CTX‐induced TA muscle regeneration. (C‐D) RT‐qPCR (C) and western blotting (D) showing the expression levels of Fgfr2 during C2C12 myogenesis. (E) The expression of Fgfr2 was quantitated by RT‐qPCR during postnatal development in the hin [file JCSM-13-696-s005.docx]

**Supplemental Figure 1-13**

**
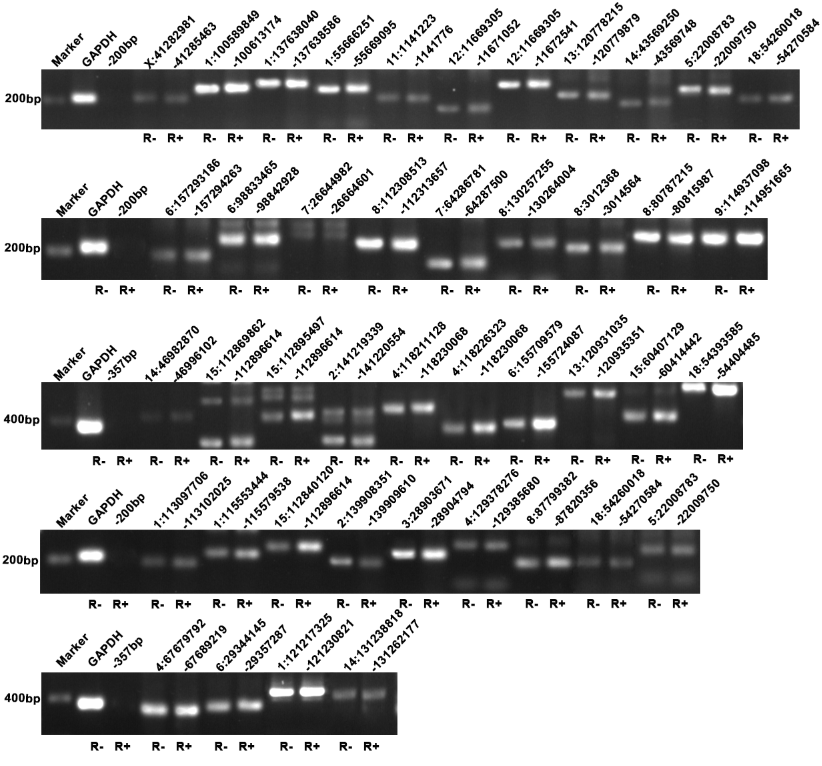
**

**Figure S1. Verification of the reliability of pig circRNAs.** Pig circRNAs were validated by PCR with reverse transcription (RT–PCR) using divergent primers following RNase R treatment.


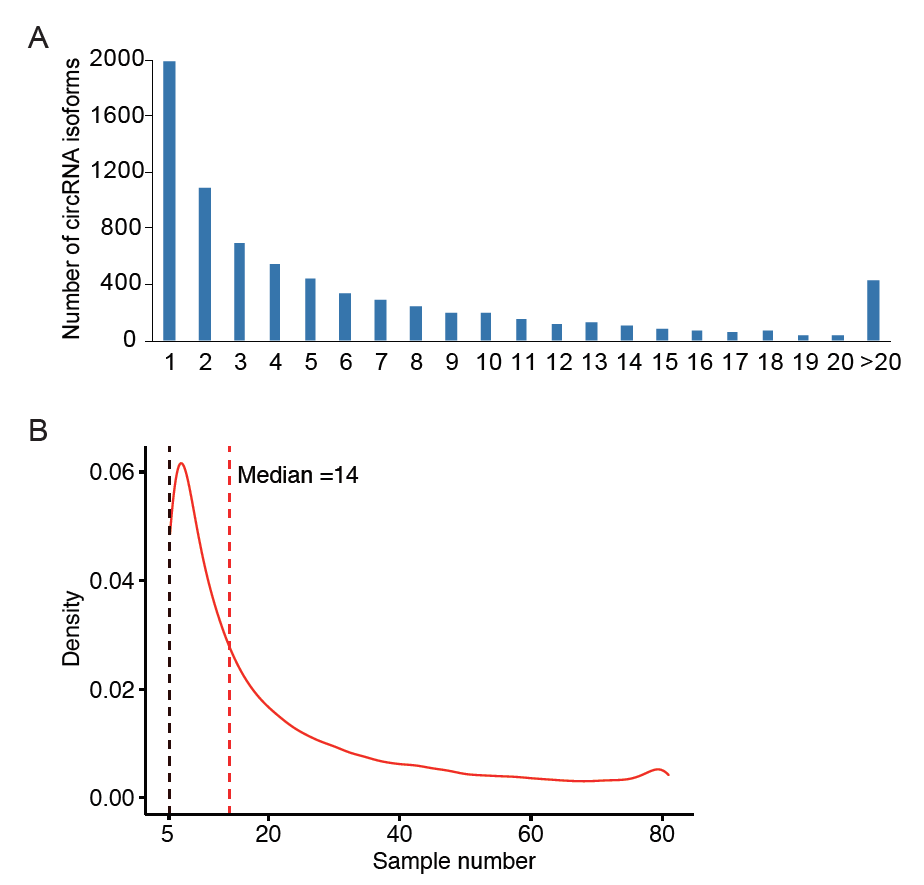


**Figure S2. The production and expression of identified circRNAs. (A)** Number of circRNAs produced from one gene. **(B)** The distribution of the sample number per circRNA expressed. **
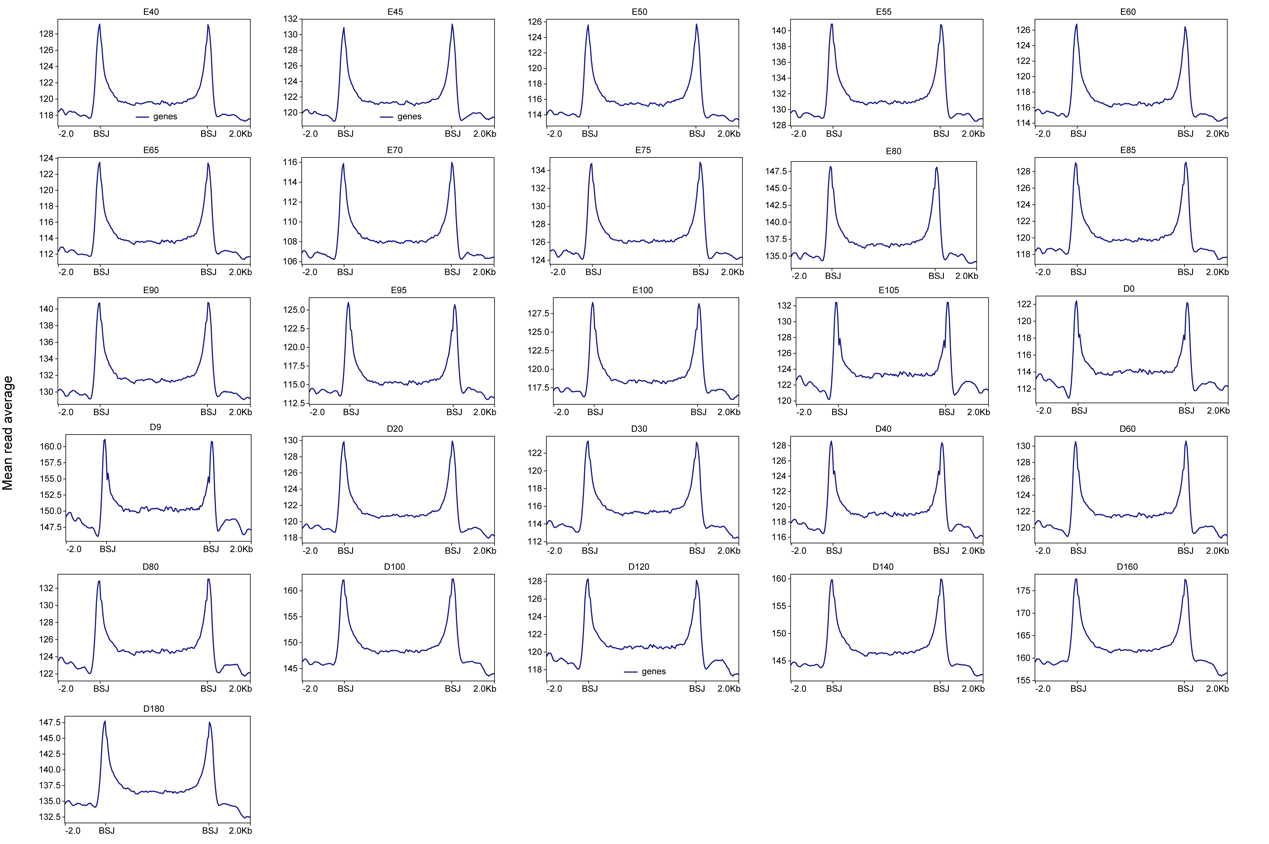
**

**Figure S3. Average read coverage of DNA methylation across gene bodies and the 2-kb regions flanking the BSJ sites of circRNAs at each developmental stage, except E33.**

**
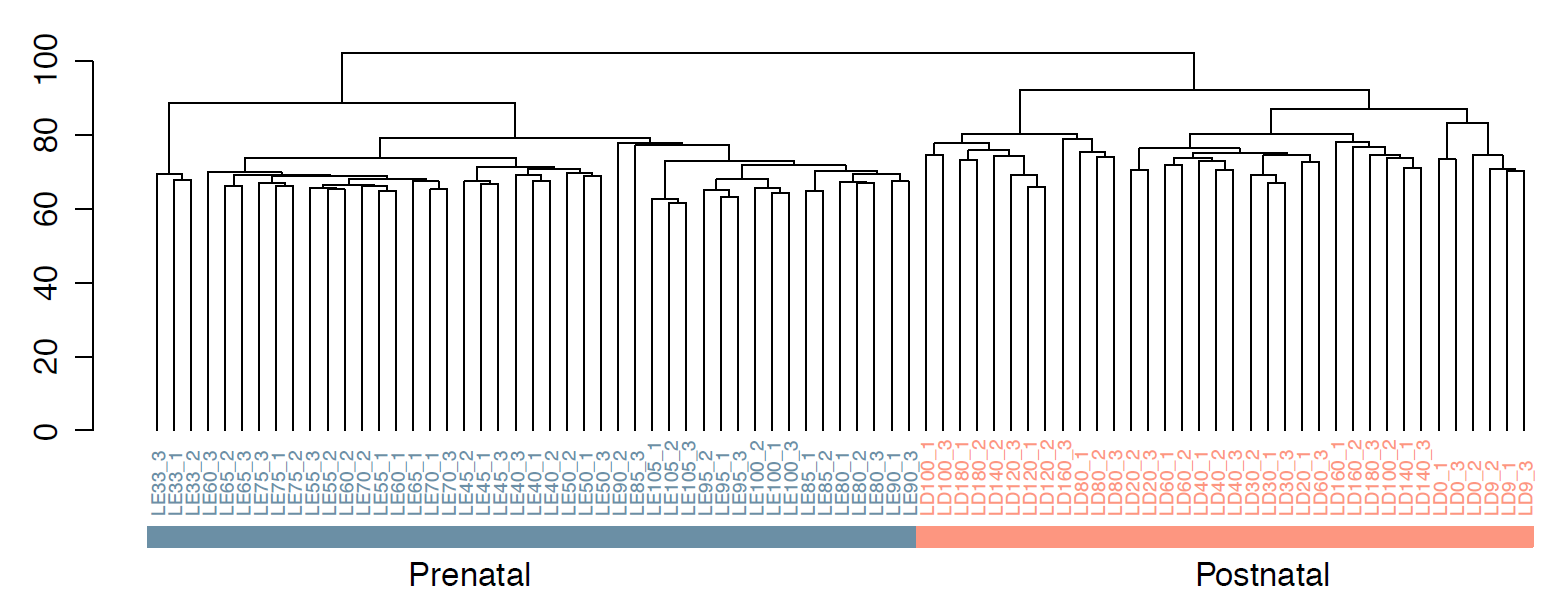
**

**Figure S4. Hierarchical cluster analysis of the skeletal muscle samples.** Clustering was performed based on the log2-transformed CPM values of 3,382 circRNAs that were expressed in at least 80% of samples using the average linkage method by the hclust function in R.


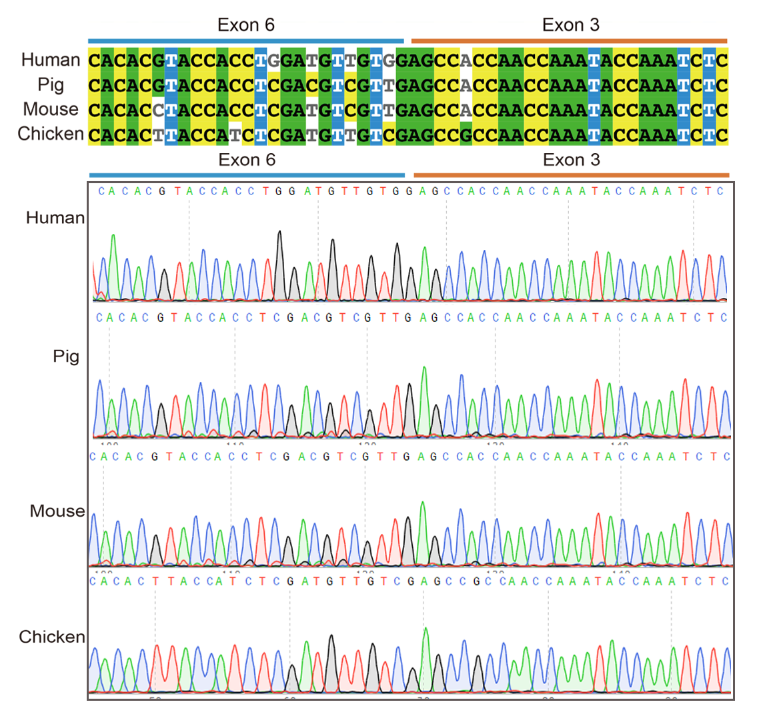


**Figure S5. Sequence alignment of the BSJ sequences of circFgfr2 in humans, mice, pigs, and chickens.** The BSJ sequences of circFgfr2 in humans (hsa-Fgfr2_0001) were downloaded from the circAltas database. The BSJ sequence of chicken circFgfr2 was obtained from a previous study [32]. The BSJ sequences of mouse and pig circFgfr2 were amplified in the present study. Sanger sequencing was performed to validate the BSJ sequences of circFgfr2 in the four species.

**
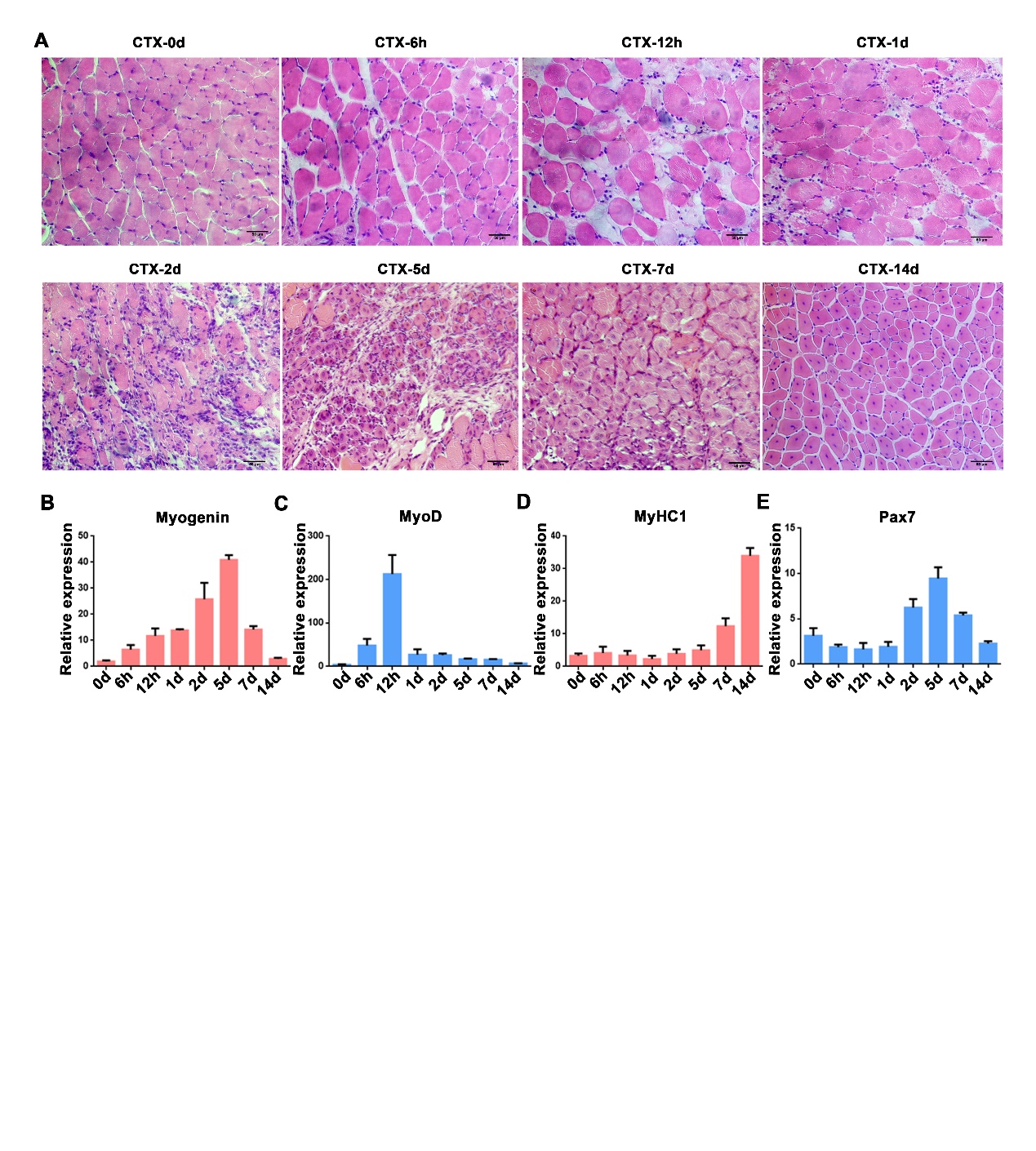
**

**Figure S6.** **Construction of the** **muscle regeneration model following CTX injection in the tibialis anterior (TA)**. **(A)** H&E staining of the cross-sections of CTX-induced TA muscle. **(B-E)** RT-qPCR showing the expression levels of *myogenin*, *MyoD*, *MyHC1,* and *Pax7* during muscle regeneration. The expression level was normalized to 18s-ribosomal RNA. N = 3–5 in each group.


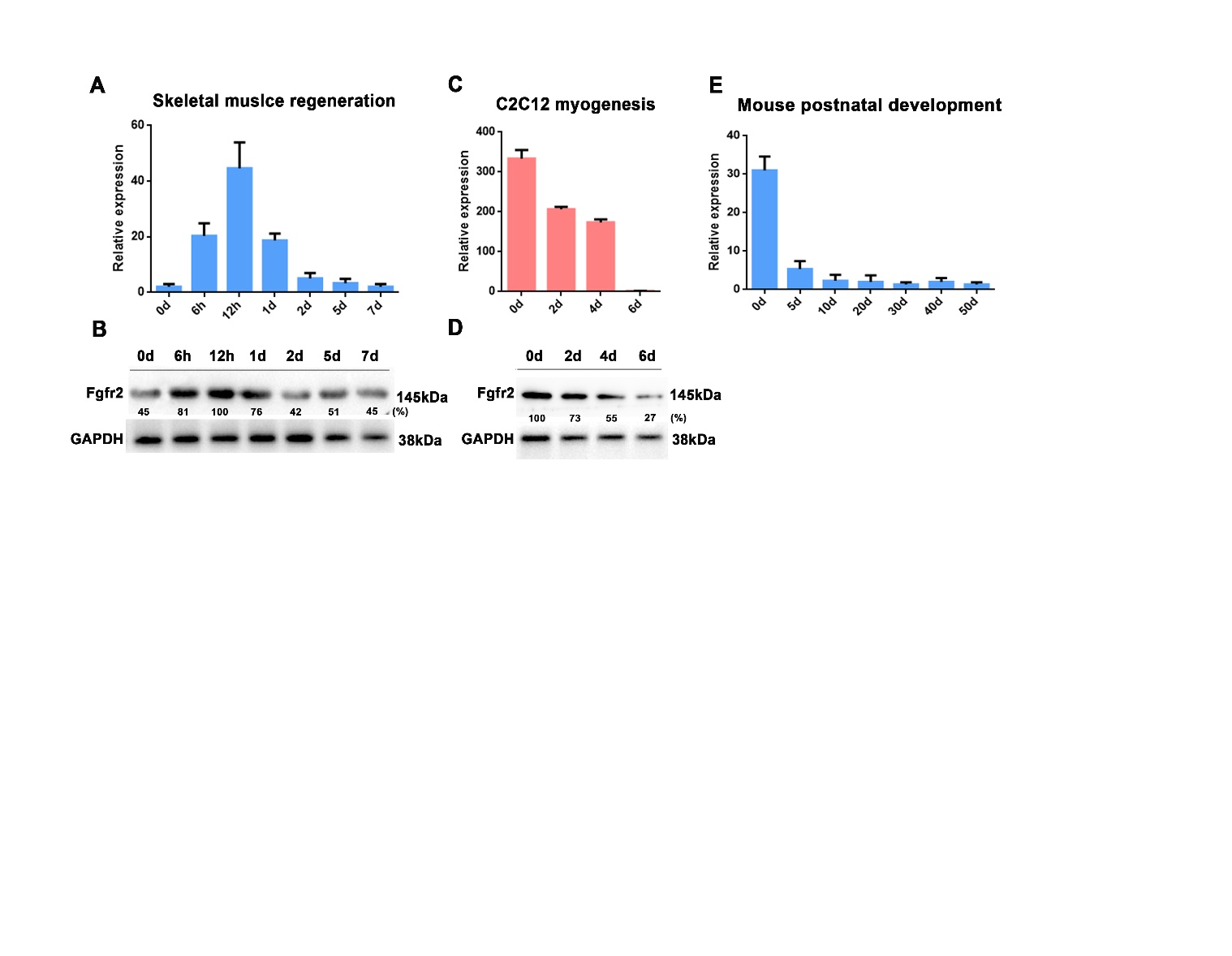


**Figure S7.** **Expression patterns of Fgfr2 in mice**. (**A-B**) The expression of Fgfr2 was quantitated by RT-qPCR (**A**) and western blotting (**B**) during CTX-induced TA muscle regeneration. (**C-D**) RT-qPCR (**C**) and western blotting (**D**) showing the expression levels of Fgfr2 during C2C12 myogenesis. (**E**) The expression of Fgfr2 was quantitated by RT-qPCR during postnatal development in the hind leg muscles of C57BL/6 mice. The expression level was normalized to 18s-ribosomal RNA. N = 3–5 in each group.


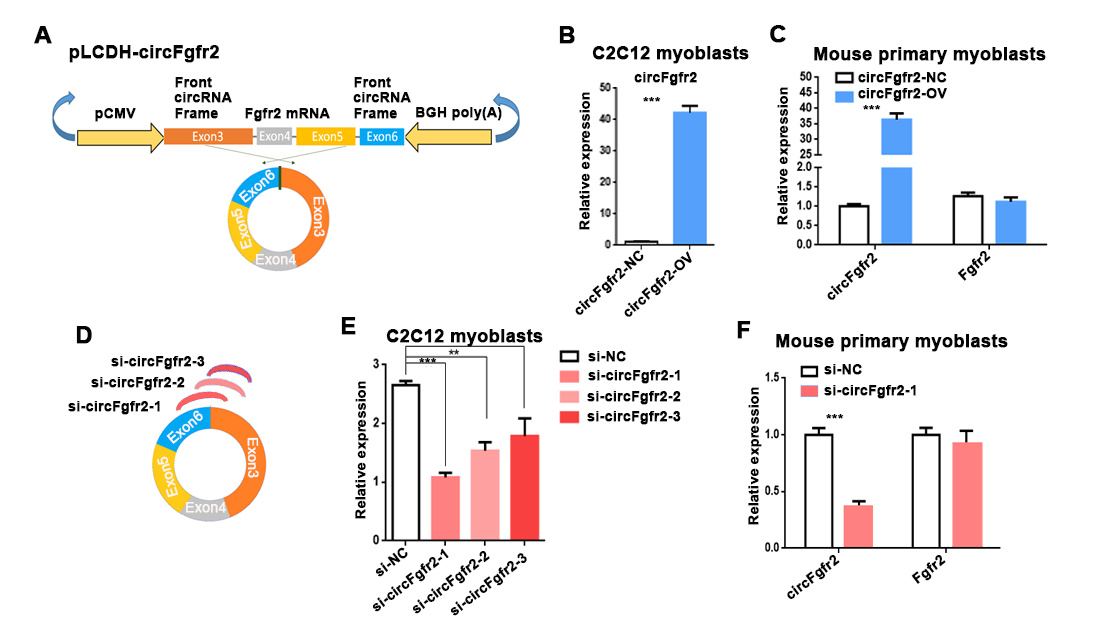


**Figure S8.** **The knockdown and overexpression of circFgfr2 in C2C12 cells and mouse primary myoblasts. (A)** Schematic diagram showing the construction of the circFgfr2-overexpression (pLCDH-circFgfr2) vector. **(B)** RT-qPCR showing the overexpression efficiency of the circFgfr2-overexpression vector in C2C12 cells. **(C)** RT-qPCR showing the effects of circFgfr2 overexpression on the expression of circFgfr2 and host gene Fgfr2 in mouse primary myoblasts. The error bars depict the mean ± S.D. of samples from three individuals. ****P* < 0.001. **(D)** Schematic diagram showing the design of three siRNA oligos against circFgfr2 (si-circFgfr2-1, -2, and -3). **(E)** RT-qPCR showing the interference efficiency of the three siRNAs in C2C12 myoblasts. **(F)** RT-qPCR showing the effect of si-circFgfr2-1 on the expression of circFgfr2 and Fgfr2 in mouse primary myoblasts. The error bars depict the mean ± S.D. of samples from three individuals. ****P* < 0.001.

­­­

**
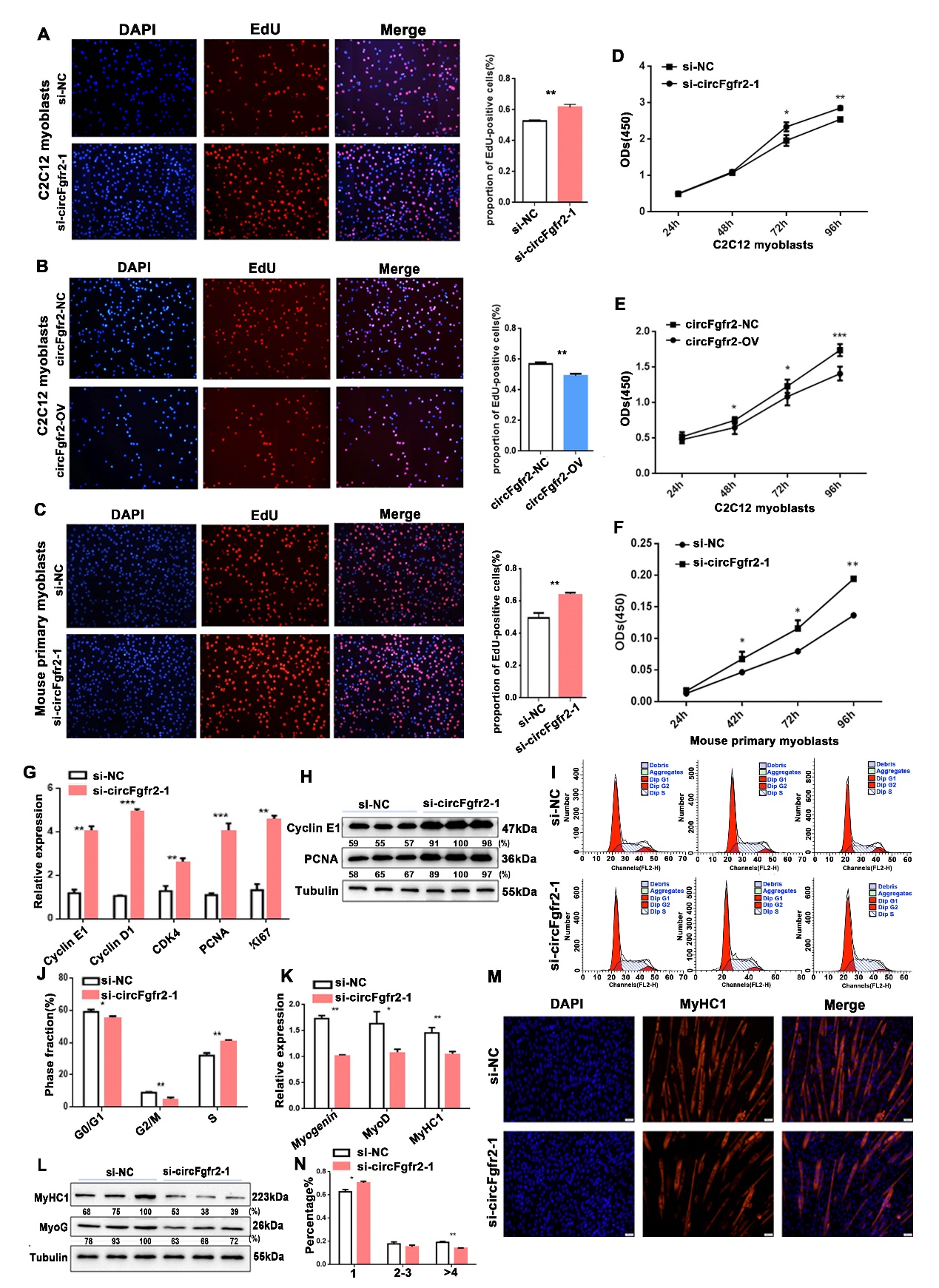
Figure S9. Knockdown of circFgfr2 promotes proliferation but prevents differentiation.** **(A-C)** Cell proliferation indices were assessed following treatment with 5-ethynyl-2′-deoxyuridine (EdU) and counted using Image J, after transfection with si-circFgfr2-1 (**A**) and circFgfr2-OV (**B**) in proliferating C2C12 myoblasts, and (**C**) si-circFgfr2-1 in proliferating mouse primary myoblasts. EdU staining (red) for positive cells; DAPI staining (blue) for cell nuclei. The error bars depict the mean ± S.D. of samples from three individuals. **P* < 0.05 **(D-F)** Cell counting kit-8 (CCK-8) assay showing the cell proliferation activity following transfection with si-circFgfr2-1 (**D**) or circFgfr2-OV (**E**) in proliferating C2C12 myoblasts, and (**F**) si-circFgfr2-1 in proliferating mouse primary myoblasts. Data are presented as the mean ± S.D. of samples from four individuals. **P* < 0.05, ***P* < 0.01, ****P* < 0.001 **(G-H)** Transfection of proliferating mouse primary myoblasts with si-circFgfr2-1 and their negative controls. The proliferation and cell cycle markers were quantitated by RT-qPCR (**G**) and western blotting (**H**). The error bars depict the mean ± S.D. of samples from three individuals. **P* < 0.05 and ***P* < 0.01. **(I-J)** The cell cycle was analyzed using flow cytometry following transfection with si-circFgfr2-1 and their negative controls in proliferating mouse primary myoblasts. The error bars depict the mean ± S.D. of samples from three individuals. **P* < 0.05 and ***P* < 0.01. **(K-L)** Transfection of mouse primary myoblasts with si-circFgfr2-1 and their negative controls. The mRNA and protein expression levels of myogenic differentiation marker genes (MyoD, myogenin, and MyHC1) were detected by RT-qPCR (**K**) and western blotting (**L**) in mouse primary myoblasts that differentiated after 4 days in vitro, respectively. The error bars depict the mean ± S.D. of samples from three individuals. **P* < 0.05 and ***P* < 0.01. (**M-N)** Immunofluorescence analysis of MyHC1 myotubes (M) and the number of nuclei per myotube was counted (N) following knockdown of circFgfr2 in mouse primary myoblasts that differentiated after 4 days in vitro; the scale bars represent 100 μm.


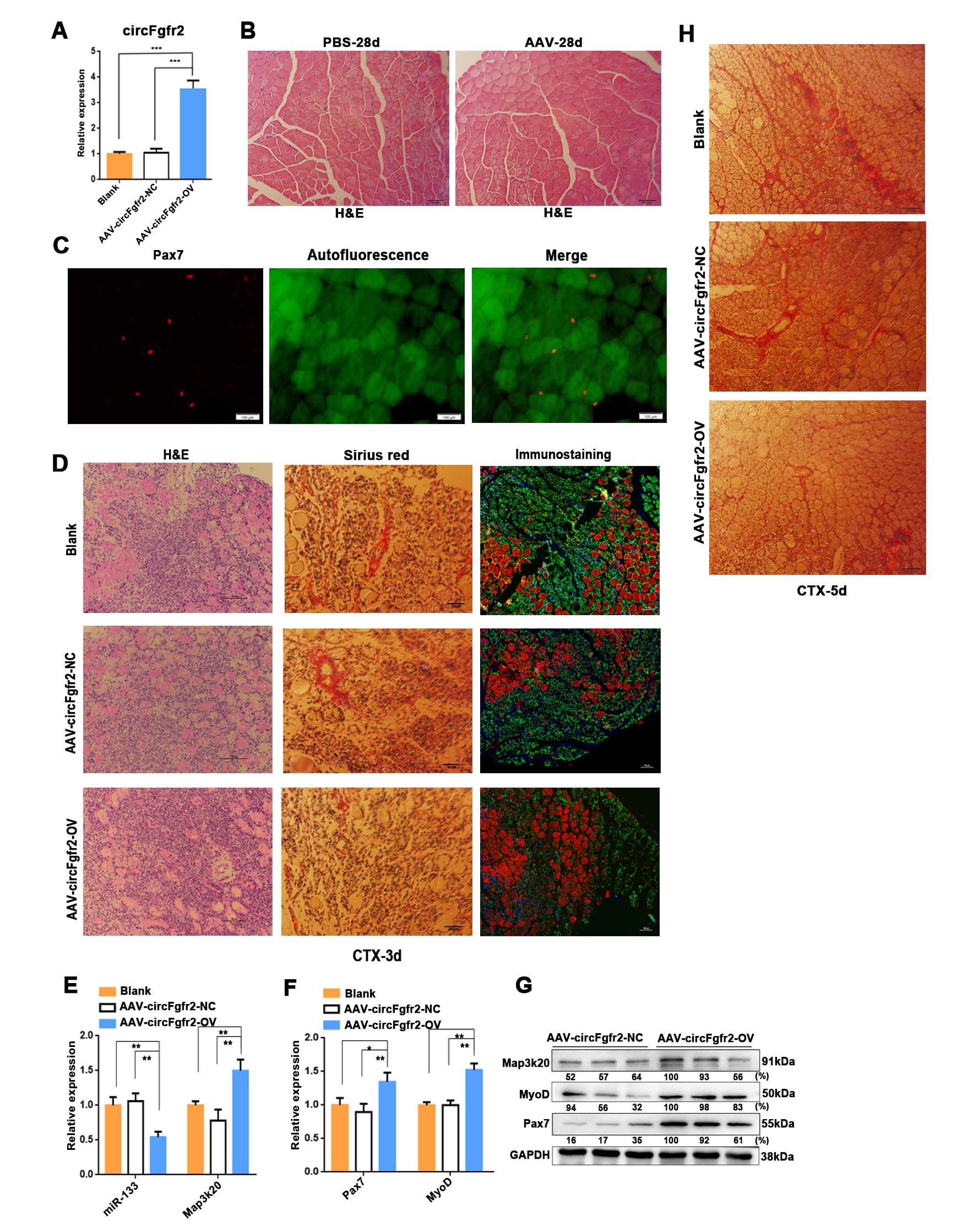


**Figure S10.** **circFgfr2 promotes skeletal muscle regeneration.** **(A-C)** RT-qPCR (**A**), H&E staining (B) and fluorescence images (**C**) showing that injection of AAV-circFgfr2-OV increased circFgfr2 expression in mouse TA muscles 28 days after adenovirus injection. The expression level was normalized to 18s-ribosomal RNA. ** *P* < 0.01 post-injury vs. baseline level; n = 5 in each group. Data are presented as the mean ± SEM. **(D)** H&E staining, Sirius Red staining and immunostaining for desmin (red) and laminin (green) of AAV-circFgfr2-OV and AAV- circFgfr2-NC TA muscles on day 3 post-CTX injury (Scale bar: 50μm in H&E staining, Sirius Red staining; 100 μm in immunostaining). (**E-F**) RT-qPCR revealing that expression of Map3k20, Pax7, and MyoD was dramatically upregulated and that of miR-133 was downregulated in AAV-circFgfr2-OV mice on day 3 post-CTX injury. The expression level was normalized to 18s-ribosomal RNA. ** *P* < 0.01 post-injury vs. baseline level; n = 3–5 in each group. Data are presented as the mean ± SEM. **(G)** Western blotting showing the expression of Map3k20, Pax7 and MyoD in AAV-circFgfr2-OV mice on day 3 post-CTX injury. (**H**) Sirius Red staining of AAV-circFgfr2-OV and AAV- circFgfr2-NC TA muscles on day 5 post-CTX injury (Scale bar: 100 μm).


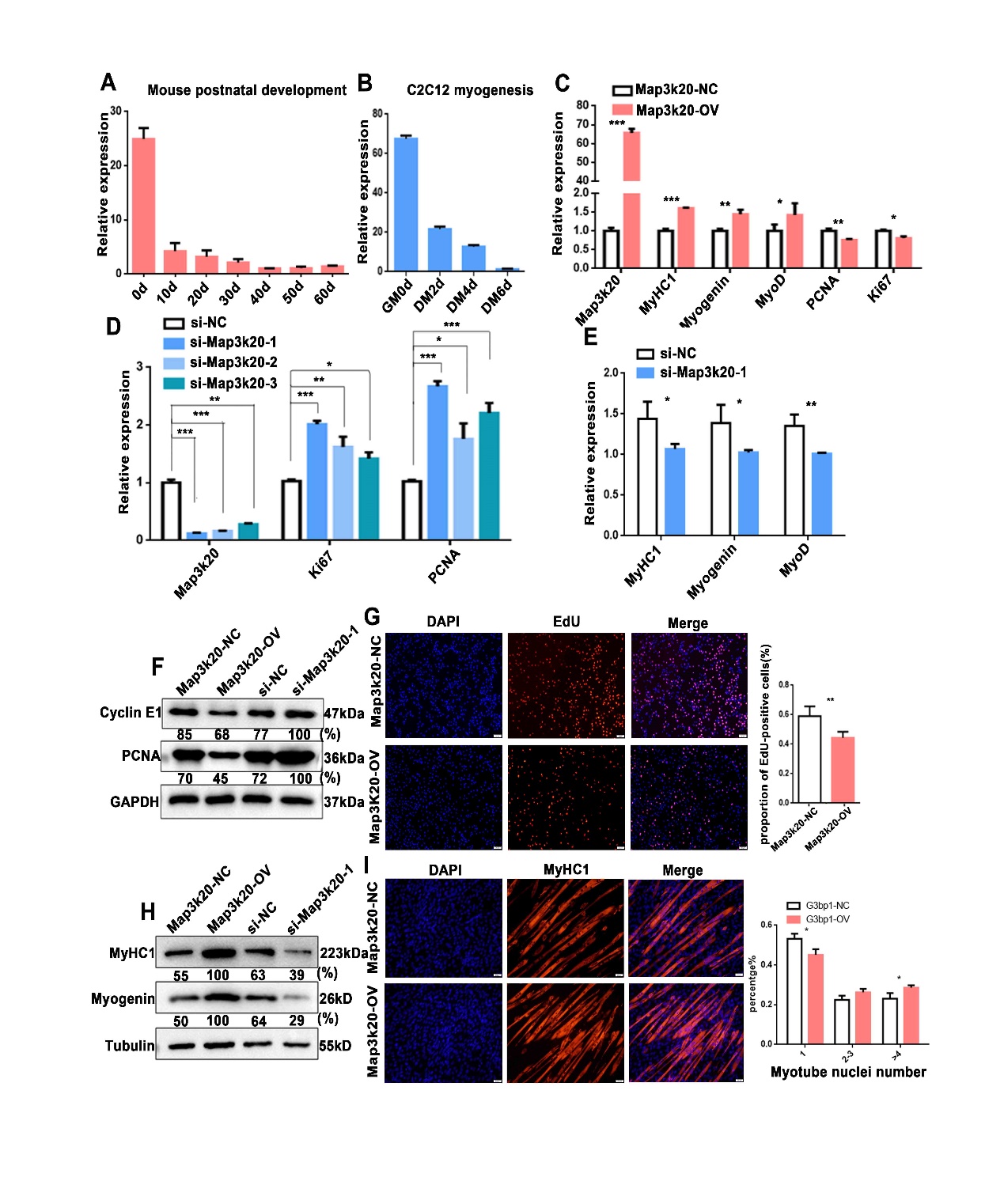


**Figure S11.** **Map3k20 promotes myoblast differentiation but inhibits proliferation in C2C12 cells.** **(A-B)** RT-qPCR showing the expression of Map3k20 during postnatal skeletal muscle development in mice (**A**) and during C2C12 myoblast myogenesis (**B**). **(C)** RT-qPCR showing the expression of proliferation markers *(PCNA and Ki67)* and myogenic differentiation marker genes *(MyoD, myogenin, and MyHC1*) following transfection with the Map3k20-overexpression vector in proliferating C2C12 cells. The error bars depict the mean ± S.D. of samples from three individuals. * *P* < 0.05, ** *P* < 0.01, *** *P* < 0.001. **(D)** RT-qPCR showing the interference efficiency of three Map3k20 siRNAs (si-Map3k20-1, -2, and -3) and their effect on the expression of Ki67 and PCNA in proliferating C2C12 myoblasts. The error bars depict the mean ± S.D. of samples from three individuals. * P < 0.05, ** P < 0.01, *** P < 0.001. **(E)** RT-qPCR showing the expression levels of myogenic differentiation marker genes (*MyoD*, *myogenin*, and *MyHC1*) following transfection with si-Map3k20-1 in C2C12 cells that differentiated after 4 days *in vitro*. The error bars depict the mean ± S.D. of samples from three individuals. * P < 0.05, ** P < 0.01. **(F)** Western blotting showing the expression levels of PCNA and Cyclin E1 in proliferating C2C12 cells following transfection of Map3k20, si-Map3k20-1, and their negative controls. **(G)** EdU assay to assess cell proliferation after transfection with Map3k20-OV or Map3k20-NC in proliferating C2C12 myoblasts. Cell proliferation indices were assessed after treatment with EdU and counted using Image J. EdU staining (red) for positive cells; Dapi staining (blue) for cell nuclei. the scale bars represent 100 μm. **(H)** Western blotting showing the expression levels of myogenin and MyHC1 after transfection with Map3k20, si-Map3k20-1, and their negative controls in C2C12 cells that differentiated after 4 days *in vitro*. **(I)** Immunofluorescence microscopy analysis of expression of MyHC1 and the number of nuclei per myotube was counted in Map3k20-overexpressing C2C12 cells that differentiated after 4 days *in vitro*. The scale bars represent 100 μm.


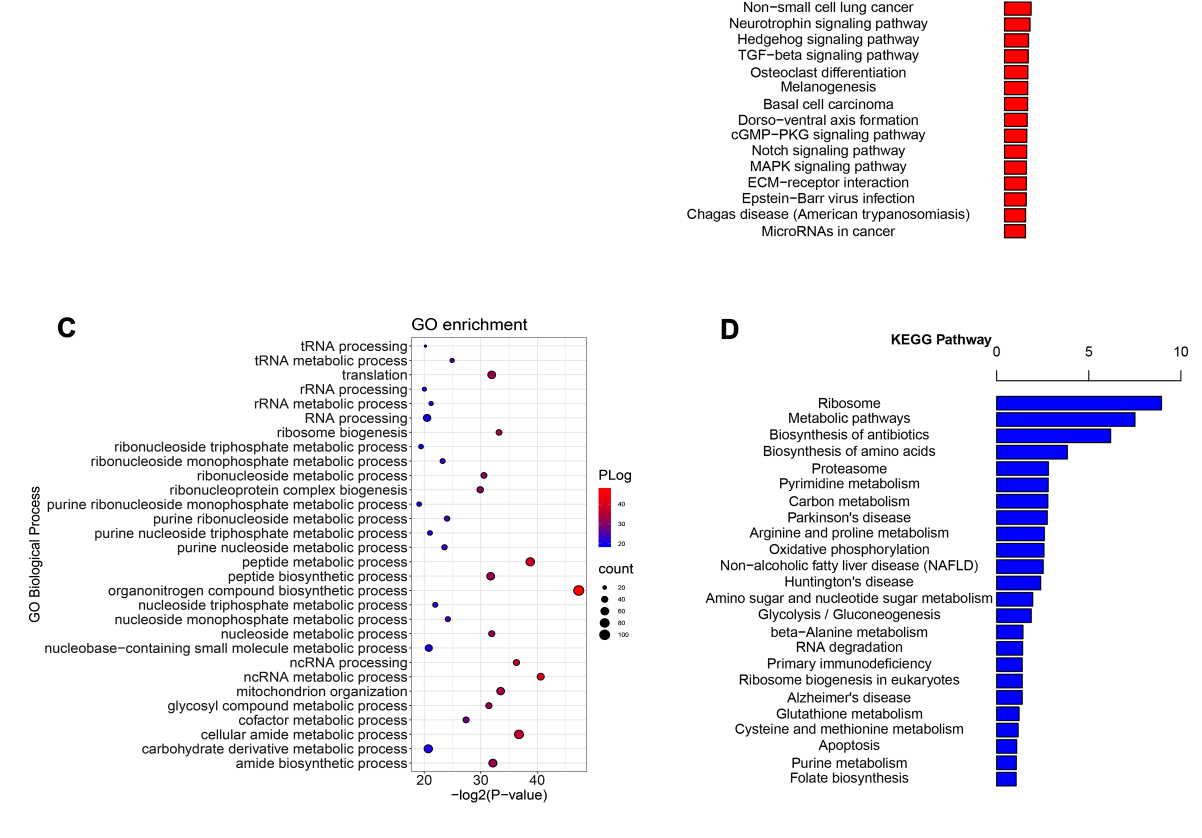

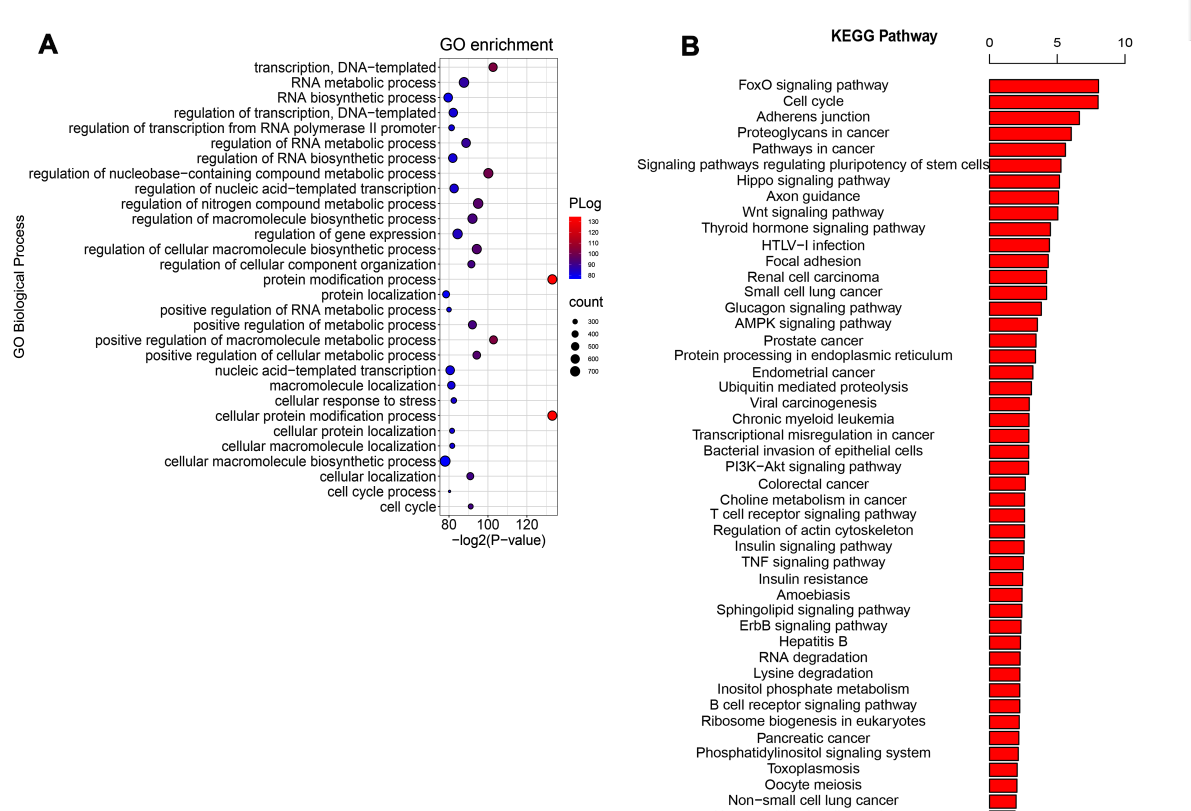
 ­

**Figure S12.** **GO and KEGG pathway analyses of differentially expressed genes in circFgfr2-overexpressing circFgfr2 myoblasts as compared with the negative control.** **(A-B)** GO (**A**) and KEGG (**B**) pathway analyses of upregulated genes. **(C-D)** GO (**C**) and KEGG (**D**) pathway analyses of downregulated genes.

**
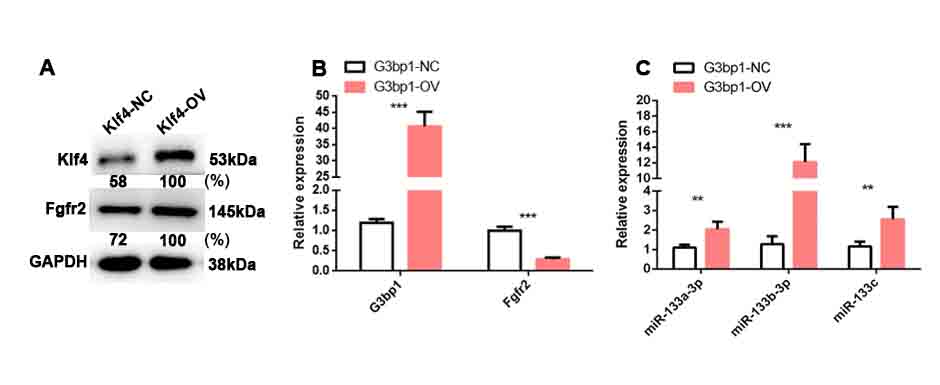
**

**Figure S13.** **Klf4 and G3bp1 modulates the transcription of circFgfr2.** (**A**)Western blotting showing the expression levels of Klf4 and Fgfr2 in C2C12 cells following transfection with Klf4-OV or Klf4-NC. (**B**) The expression level of G3bp1 and Fgfr2 in G3BP1-overexpressing C2C12 cells. The error bars depict the mean ± S.D. of samples from three individuals. *** *P* < 0.001. **(C)** The expression level of miR-133 family in G3BP1-overexpressing C2C12 cells. The error bars depict the mean ± S.D. of samples from three individuals. ** *P* < 0.01, *** *P* < 0.001.
