# Supplementary material for: circRNAome profiling reveals circFgfr2 regulates myogenesis and muscle regeneration via a feedback loop
Source: J Cachexia Sarcopenia Muscle. 2021 Nov 22;13(1):696–712. doi: 10.1002/jcsm.12859 (PMC8818660; doi:10.1002/jcsm.12859)
Supplement: Supplementary file 2 — Data S1. Detail methods used in the present study. [file JCSM-13-696-s003.docx]

**Supplemental Methods**

**Identification and analysis of circRNAs using RNA-seq data**

The Ribo-zero strand-specific RNA-seq data used for circRNA identification were obtained from our previous studies (GEO accession number GSE157044) [1, 2]. The transcriptome data were generated from the skeletal muscle of Landrace pigs at 27 developmental stages covering the life cycle from embryonic day 33 (E33) to postnatal day 180 (D180), and consisted of 8.2 G reads that were sequenced as 150 bp paired-end reads. The corresponding whole-genome bisulfite sequencing (WGBS) data during these 27 developmental stages (GEO accession number GSE157043) were used to evaluate the methylation level across the circRNAs.

Two detection algorithms, CIRI2 [3] and find_circ [4], were used to identify novel circRNAs using default parameters. circRNAs that were detected by both programs and contained at least 2 unique back-spliced reads were retained. High-confidence circRNAs were required to be expressed in a minimum of 5 distinct samples. The expression level of each circRNA in each sample was quantitated by calculating the counts per million mapped reads (CPM) using CIRIquant (v1.1) [5]. Differentially expressed circRNAs between any two developmental stages were identified by CIRIquant [5] using a threshold of FDR ≤0.05 and an absolute fold change ≥1. The full-length sequence of each circRNA was extracted from the pig reference genome (NCBI Sscrofa11.1) [6] using R package FcircSEC (v1.0.0) [7]. The known circRNAs in pigs, mice, and humans were downloaded from the circAltas database [8].

The methylation profiles of the upstream 2k, downstream 2k, or gene body regions of the circRNAs were generated by computeMatrix and plotProfile in the deepTools suite (v3.3.0) [9]. Conserved circRNAs across species were identified using the criterion of ≥70% sequence identity between the BSJ sequences (extended 25 base pairs (bp) on either end of the splice site) originating from homologous genes using UCSC BLAT (https://genome.ucsc.edu/cgi-bin/hgBlat). Co-expression circRNA analysis was performed using the weighted gene co‑expression network (WGCNA) package [10] in R 3.6.3 with the best soft-thresholding 6.

**Animals studies**

C57BL/6 male mice were purchased from the Charles River Labs (Beijing, China) and housed up to five animals per cage in the animal facilities of the Institute of Animal Sciences, Chinese Academy of Agricultural Sciences. Animals were subjected to a 12/12-h light/dark cycle and had access to food and water *ad libitum.* All animal procedures were performed according to the protocols of the Chinese Academy of Agricultural Sciences and the Institutional Animal Care and Use Committee. No drug tests were carried out. Normal TA muscles were collected on postnatal days 0, 5, 10, 20, 30, 40, 50, and 60 with at least three biological replicates at each time point.

For CTX administration, adenoviruses expressing circFgfr2 (AAV-circFgfr2-OV) or the negative control (AAV-circFgfr2-NC) (10^10^–10^11^ p.f.u./mL) were injected into the TA muscles of 6-week-old C57BL/6 mice. After 28 days, the mice were injected with 100 μL CTX (10 μM) at the same place. Mice were sacrificed and TA muscles were collected at 0h, 6h, and 12h, and on days 1, 2, 3, 5, 7, and 14 post-CTX injury. One sample at each time point was fixed in 4% paraformaldehyde for histology and immunohistochemistry. The other samples were frozen in liquid nitrogen and stored at −80°C for RNA and protein extraction.

**Cell isolation and culture**

To isolate mouse primary myoblasts, the TA muscles of newborn mice were collected. The hind limb muscles were minced and digested with 0.2% type II collagenase (Sigma) for 30 min, followed by 0.25% Trypsin-EDTA (Gibco) for 20 min. Each sample was consecutively filtered through a 70-μm cell strainer and the cell suspension was centrifuged at 600 × g. The primary myoblasts were cultured in Ham’s F10 nutrient medium (Gibco) supplemented with 20% FBS (Gibco) and 5 ng/mL bFGF. HEK293T and C2C12 cells were cultured in DMEM (Gibco) supplemented with 10% FBS in a humidified incubator with 5% CO_2_ at 37°C. To induce myogenic differentiation, mouse primary myoblasts and C2C12 cells were incubated in DMEM supplemented with 2% heat-inactivated horse serum (Gibco).

**Plasmid construction and RNA interference**

To construct the circFgfr2-overexpression vector, the full-length sequence of mouse circFgfr2 was cloned into the pLCDH-ciR vector (Geenseed Biotech, Guangzhou, China), and the mock vector lacking the circFgfr2 sequence was used as a negative control. To knock down circFgfr2, three siRNAs (si-circFgfr2-1, -2, -3) targeting the BSJ of circFgfr2 and a negative control (siRNA-NC) were synthesized (Ribobio Biotech, Guangzhou, China). The siRNA, si-circFgfr2-1, with the highest efficiency was chosen for subsequent analysis. mmu-miR-133 mimics and inhibitors were bought from Ribobio Biotech (Guangzhou, China).

To construct the Fgfr2 promoter luciferase reporter plasmid, we cloned three continuous regions of mouse Fgfr2 promoter(B1-B3), B1 contained the sequence from -1138bp to -865 bp，B2 contained the sequence from -865bp to -576 bp, B3 contained the sequence from -576bp to -120 bp. These regions were subcloned into the pGL3-Basic vector (Promega) using the Sac1/XhoI sites.

The overexpression vectors for G3bp1, Map3k20, and Klf4 were constructed in pcDNA3.1, and siRNAs against these genes were purchased from Ribobio Biotech (Guangzhou, China). The siRNA and shRNA sequences used in the present study are listed in Table S1.

**Luciferase reporter assay**

The plasmid (pGL3-Basic) containing the circRNA and Map3k20 3'UTR corresponding to the miR-133, were generated by PCR amplification and subcloned into the pGL3-Basic luciferase reporter vector (Promega), including wild-type and mutant type.

For the Fgfr2 promoter activity assay, we cotransfected HEK293T cells with Fgfr2 promoter luciferase reporter vector (pGL3-B1/pGL3-B2/pGL3-B3) and the overexpression vectors for Klf4 or their empty vector. We chose HEK293T cells cotransfected with empty pGL3-Basic vector and empty pcDNA3.1 vector as negative controls.

Luciferase assays were performed 48 hours after transfection using the Dual Luciferase Reporter Assay System (Promega). Each transfected well was assayed in triplicate. Firefly luciferase activity was normalized to Renilla luciferase activity for each transfected well.

**circRNA microarray analysis**

Total RNA was isolated from C2C12 myoblasts, during proliferation (GM) and 4 days after induction of differentiation (DM), using TRIzol reagent according to the manufacturer's protocol. circRNAs were enriched by removing linear RNAs using RNase R (Epicentre), and the enriched circular RNAs were amplified and transcribed into fluorescently labeled cDNA utilizing a random priming method (Arraystar Super RNA Labeling Kit; Arraystar). The circRNA microarray was analyzed using the Arraystar Mouse circRNA Array V2 (Arraystar, Rockville, MD, USA). Four biological replicates were performed in each group. The Agilent Feature Extraction software (version 11.0.1.1) was used to analyze the acquired array images. After quantile normalization of the raw data, the differentially expressed circRNAs with statistical significance between DM and GM were identified using a Student's *t*-test with a cut-off value for FDR ≤ 0.05 and an absolute fold change ≥ 2.

**RNA-sequencing analysis**

The RNA-seq libraries were prepared using the NEBNext Ultra RNA Library Prep Kit for Illumina (NEB, USA) according to the manufacturer’s instructions, and were sequenced on an Illumina NovaSeq platform to generate 150-bp paired-end reads. Three biological replicates were performed in each group. Following removal of reads containing adapters and poly-N and those of low-quality, the clean reads were aligned with the mouse reference genome (Ensembl GRCm38) using HISAT2 (version 2.0.5). The gene annotation file was downloaded from the Ensembl website (release_M25). HTSeq (version 0.12.4) was used to count the read numbers mapped to each gene [11]. DESeq2 (version 1.22.2) [12] was then applied to determine differentially expressed genes with a cut-off value for FDR ≤ 0.05 and an absolute fold change ≥ 1. GO and KEGG enrichment analyses of the differentially expressed genes were performed using the DAVID Bioinformatics Resources 6.8 (<https://david.ncifcrf.gov/>) [13].

**Single-cell RNA-sequencing analysis**

Five days after CTX injection, TA muscles from the AAV-circFgfr2-OV and AAV-circFgfr2-NC groups were isolated and digested with collagenase I (800 U/mL) and Dispase II (11 U/mL) for 30 min. The digestive residue was screened and filtered using a 40-µm cell strainer, after which it was washed twice with DPBS (+2% FBS). Subsequently, library construction and single-cell RNA-seq were performed according to the instructions of the Chromium Next GEM Single Cell 3 Reagent Kit v3.1. Sequencing reads were processed by Cell Ranger version 3.0.1 (10x Genomics, Pleasanton, CA) using the mouse reference transcriptome mm10. Based on the gene expression matrix, downstream analysis was carried out using R version 3.6.2 (2019-12-12). Quality control, filtering, data clustering, data visualization, and differential expression analysis were performed using Seurat version 2.3.4 R package [14] with some custom modifications to the standard pipeline.

**Cell proliferation and cell cycle assay**

To examine cell proliferation, 10 μL Cell Counting Kit-8 (CCK-8) reagent (Dojindo) was added to each well of a 96-well plate containing cells and incubated at 37°C for 45 min. The absorbance of each sample at 450 nm was measured using a microplate reader. In addition, cell proliferation was assessed using the Cell-Light EdU DNA cell proliferation kit (Ribobio) according to the manufacturer’s instructions. Five independent replicates were performed for each treatment group. Flow cytometry analysis of the cell cycle was performed on a BD Accuri C6 flow cytometer (BD Biosciences, San Jose, CA, USA) as previously described [15], and data were processed using the FlowJo7.6 software (Treestar Incorporated, Ashland, OR, USA).

**RNA preparation, RT-PCR, and RT-qPCR**

Total RNA was extracted from skeletal muscle and cells using TRIzol reagent (Invitrogen) according to the manufacturer’s instructions, and cDNAs were prepared using reverse transcriptase (Thermo Fisher Scientific). Oligo(dT) primers were used for coding genes and random primers were used for circRNAs. RNAs from the nucleus and cytoplasm of C2C12 myoblasts were separated using a Cytoplasmic and Nuclear RNA Purification Kit (Norgen Biotek, Thorold, Canada) following the manufacturer’s instructions. For RNase R treatment, 1 mg total RNA was incubated for 15 min at 37°C with 4 U/μg RNase R and subsequently purified using an RNeasy MinElute Cleaning Kit (QIAGEN). RT-PCR and Sanger sequencing were performed to validate circRNAs using divergent primers. Analysis of mRNA, miRNA, and circRNA expression was performed with SYBR Green Master Mix (ABI). RT-qPCR data were analyzed using the ^ΔΔ^Ct method, and individual gene expression was normalized to GAPDH expression or 18s RNA expression. The primer sequences used in the present study are listed in Table S1.

**Western blotting**

Total protein was extracted from tissues and cells and used as previously described [1, 16]. Proteins were separated by SDS-polyacrylamide gel electrophoresis (SDS-PAGE) and transferred to nitrocellulose membrane. The membranes were blocked with 5% skim milk for 1.5 h at room temperature, and subsequently probed with primary antibodies overnight at 4℃. The following dilutions were used for each antibody: MyoG (1:1000; Proteintech), MyHC1 (1:1000; Dshb), Ki67 (1:1000; abcam), MYOD (1:1000; Proteintech), Pax7 (1:1000; Proteintech), GAPDH (1:1000; Proteintech), tubulin (1:1000; Proteintech), MKK7 (1:1000; CST), p-MKK7 (1:1000; CST), JNK (1:1000; CST), and p-JNK (1:1000; CST). The following day, the membranes were washed with PBS-Tween and incubated for 30 min with horseradish peroxidase-conjugated secondary antibodies (Proteintech). Protein bands were detected after treatment with SuperSignal West Femto agent (Thermo Scientific).

**Histology**

Hematoxylin and eosin (H&E) staining and Picro-sirius red staining were used for descriptive analysis of TA muscle regeneration. TA muscles were either paraformaldehyde-fixed, dehydrated, and embedded in paraffin. The specimens were sectioned longitudinally (8μm). The slides were stained with hematoxylin and eosin (H&E) or Sirius Red[17]. The images were visualized and captured with the Olympus BX51-P microscope.

**RNA fluorescence *in situ* hybridization assay (FISH)**

The RNA-FISH assay was performed in C2C12 myoblasts following the manufacturer’s instructions (GEFAN). The probe sequence for circFgfr2 is 5'-ATTTGGTTGGTGGCTCAACGACATCGAGGTGGTAGGTGTGGTTGA-3', and that for Map3k20 is 5'- ACCTCCCTTGTTAGCATCTCCCAGAGAACCACACCATAGGAATAC-3’. Briefly, cells were seeded onto cover-glass in 6-well plates, cultured to 70–80% confluence, and fixed. Following treatment with 0.1% Triton X-100, cells were incubated with 20 mg/mL probes overnight at 37℃. Nuclei was counterstained with DAPI. Images were acquired using an FV1200 laser confocal microscope (Olympus).

**Immunohistochemistry and immunostaining**

Frozen sections were fixed in acetone for 10 min on ice and cells were fixed on coverslips with 4% paraformaldehyde for 15 min, washed with PBS, and treated with 0.3% Triton X-100/PBS at room temperature for a further 20 min. Sections or cells were subsequently incubated with 5% (vol/vol) goat serum/PBS and 10% BSA at room temperature for 1 h, followed by incubation with primary antibodies (diluted in 5% goat serum/PBS/0.1% Triton X-100) for 2 h. Sections or cells were then washed in PBS and incubated with secondary antibodies for 1 h. Primary and secondary antibodies were as follows: anti-Desmin (1:100, Proteintech), anti-Pax7 (1:100, Proteintech), anti-MHC1, anti-Laminin (1:200, Abcam), Alexa Fluor 594 goat anti-mouse IgG (1:400, Proteintech), and Alexa Fluor 488 goat anti-rabbit IgG (1:400, Proteintech).

**RNA immunoprecipitation (RIP)**

C2C12 cells were crosslinked with 1% formaldehyde and lysed in RIPA buffer (50 mM Tris, pH 7.4, 150 mM NaCl, 1 mM EDTA, 0.1% SDS, 1% NP-40, 0.5% sodium deoxycholate, 0.5 mM DTT, 1 mM PMSF, 1× proteinase inhibitor cocktail and 1% Rnase Out). The lysate was incubated with an G3bp1 antibody or IgG control overnight. The RNA/protein complex was recovered using protein G Dynabeads™ and washed with RIPA buffer several times. Following digestion with proteinase K, RNA was recovered using TRIzol and analyzed by RT-qPCR.

**Chromatin immunoprecipitation (ChIP)**

Chromatin immunoprecipitation was performed using a ChIP Assay Kit (EMD Millipore Corporation, Billerica, MA, USA). The crosslinking reaction was terminated using glycine in C2C12 cells treated with 1% formaldehyde. Subsequently, samples were lysed on ice for 10 min in lysis buffer, and chromatin shearing was performed by 10 cycles of sonication for 10 sec with incubation on ice for 10 seconds in-between. An anti-Klf4 antibody (Abcam) was added to form the antibody–target protein–DNA complex and protein A-Sepharose beads were used to immunoprecipitate the complex. After washing and reversing the crosslinking, the enriched DNA was purified and subsequently examined by RT-qPCR.

**Acknowledgements**

The authors of this manuscript certify that they comply with the ethical guidelines for authorship and publishing in the Journal of Cachexia, Sarcopenia and Muscle[18].

**References**

1. Yang, Y., et al., *Developmental atlas of the RNA editome in Sus scrofa skeletal muscle.* DNA Res, 2019. **26**(3): p. 261-272.

2. Yang, Y., et al., *A comprehensive epigenome atlas reveals DNA methylation regulating skeletal muscle development.* Nucleic Acids Research, 2021: p. gkaa1203.

3. Gao, Y., J. Zhang, and F. Zhao, *Circular RNA identification based on multiple seed matching.* Briefings in bioinformatics, 2018. **19**(5): p. 803-810.

4. Memczak, S., et al., *Circular RNAs are a large class of animal RNAs with regulatory potency.* Nature, 2013. **495**(7441): p. 333-8.

5. Zhang, J., et al., *Accurate quantification of circular RNAs identifies extensive circular isoform switching events.* Nature communications, 2020. **11**(1): p. 1-14.

6. Warr, A., et al., *An improved pig reference genome sequence to enable pig genetics and genomics research.* GigaScience, 2020. **9**(6): p. giaa051.

7. Hossain, M.T., et al., *FcircSEC: An R Package for Full Length circRNA Sequence Extraction and Classification.* International Journal of Genomics, 2020. **2020**: p. 9084901.

8. Wu, W., P. Ji, and F. Zhao, *CircAtlas: an integrated resource of one million highly accurate circular RNAs from 1070 vertebrate transcriptomes.* Genome Biology, 2020. **21**(1): p. 1-14.

9. Ramirez, F., et al., *deepTools: a flexible platform for exploring deep-sequencing data.* Nucleic Acids Res, 2014. **42**(Web Server issue): p. W187-91.

10. Langfelder, P. and S. Horvath, *WGCNA: an R package for weighted correlation network analysis.* BMC bioinformatics, 2008. **9**(1): p. 559.

11. Anders, S., P.T. Pyl, and W. Huber, *HTSeq-a Python framework to work with high-throughput sequencing data.* Bioinformatics, 2015. **31**(2): p. 166-169.

12. Love, M.I., W. Huber, and S. Anders, *Moderated estimation of fold change and dispersion for RNA-seq data with DESeq2.* Genome Biology, 2014. **15**(12): p. 550.

13. Huang, D.W., B.T. Sherman, and R.A. Lempicki, *Systematic and integrative analysis of large gene lists using DAVID bioinformatics resources.* Nature Protocols, 2009. **4**(1): p. 44-57.

14. Butler, A., et al., *Integrating single-cell transcriptomic data across different conditions, technologies, and species.* Nat Biotechnol, 2018. **36**(5): p. 411-420.

15. Chen, X., et al., *A novel circular RNA generated by FGFR2 gene promotes myoblast proliferation and differentiation by sponging miR-133a-5p and miR-29b-1-5p.* Cells, 2018. **7**(11): p. 199.

16. Yang, Y.L., et al., *Wnt antagonist, secreted frizzled-related protein 1, is involved in prenatal skeletal muscle development and is a target of miRNA-1/206 in pigs.* Bmc Molecular Biology, 2015. **16**: p. 4.

17. de Oliveira, F., et al., *Cyclooxygenase-2 expression in skeletal muscle of knockout mice suffering Duchenne muscular dystrophy.* Histochem Cell Biol, 2013. **139**(5): p. 685-9.

18. von Haehling, S., et al., *Ethical guidelines for publishing in the Journal of Cachexia, Sarcopenia and Muscle: update 2019.* J Cachexia Sarcopenia Muscle, 2019. **10**(5): p. 1143-1145.
